# Transmural Distribution of Coronary Perfusion and Myocardial Work Density Due to Alterations in Ventricular Loading, Geometry and Contractility

**DOI:** 10.3389/fphys.2021.744855

**Published:** 2021-11-24

**Authors:** Lei Fan, Ravi Namani, Jenny S. Choy, Ghassan S. Kassab, Lik Chuan Lee

**Affiliations:** ^1^Department of Mechanical Engineering, Michigan State University, East Lansing, MI, United States; ^2^California Medical Innovations Institute, San Diego, CA, United States

**Keywords:** coronary flow, cardiac work, cardiac mechanics, myocardial work density-perfusion mismatch, computational modeling

## Abstract

Myocardial supply changes to accommodate the variation of myocardial demand across the heart wall to maintain normal cardiac function. A computational framework that couples the systemic circulation of a left ventricular (LV) finite element model and coronary perfusion in a closed loop is developed to investigate the transmural distribution of the myocardial demand (work density) and supply (perfusion) ratio. Calibrated and validated against measurements of LV mechanics and coronary perfusion, the model is applied to investigate changes in the transmural distribution of passive coronary perfusion, myocardial work density, and their ratio in response to changes in LV contractility, preload, afterload, wall thickness, and cavity volume. The model predicts the following: **(1)** Total passive coronary flow varies from a minimum value at the endocardium to a maximum value at the epicardium transmurally that is consistent with the transmural distribution of IMP; **(2)** Total passive coronary flow at different transmural locations is increased with an increase in either contractility, afterload, or preload of the LV, whereas is reduced with an increase in wall thickness or cavity volume; **(3)** Myocardial work density at different transmural locations is increased transmurally with an increase in either contractility, afterload, preload or cavity volume of the LV, but is reduced with an increase in wall thickness; **(4)** Myocardial work density-perfusion mismatch ratio at different transmural locations is increased with an increase in contractility, preload, wall thickness or cavity volume of the LV, and the ratio is higher at the endocardium than the epicardium. These results suggest that an increase in either contractility, preload, wall thickness, or cavity volume of the LV can increase the vulnerability of the subendocardial region to ischemia.

## Introduction

Myocardial blood flow across the left ventricular (LV) wall is affected by many factors that depend on LV mechanics. These factors include the compressive intramyocardial (extravascular) pressure (IMP), perfusion pressure, and myocardial work density, which is hypothesized to regulate coronary blood flow (Goodwill et al., 2018). The IMP is generated by ventricular contraction (Namani et al., 2020) which varies from its maximum value at the endocardium to its minimum value at the epicardium (Downey et al., 1975; Algranati et al., 2010). This trend is widely used to explain the transmural distribution of myocardial blood flow across the LV wall under maximally dilated conditions, where flow is small at the endocardium and large at the epicardium (Bache et al., 1981a). When taken with the findings that total coronary flow over one cardiac cycle at the endocardium is slightly higher than epicardium under resting condition (with flow regulation), coronary flow reserve (CFR, ratio of maximally dilated flow to regulated flow) is lower at the endocardium than epicardium (Hoffman, 1987). As a result, CFR is exhausted first at the endocardium (Gallagher et al., 1980) compared to epicardium, which can explain the observation that the endocardium is more vulnerable to ischemia than the epicardium (Algranati et al., 2011).

A mismatch or imbalance between regional myocardial demand and supply is one of the hallmarks of many heart diseases, especially in ischemic heart failure (Perin et al., 2003) where insufficient blood is supplied to the heart muscle that results in heart dysfunction (Bassingthwaighte et al., 2012; Heusch, 2019). Changes in ventricular mechanics [e.g., LV contractility (Duncker and Bache, 2008)], its function [e.g., preload (Ellis and Klocke, 1980) and afterload (Hoffman and Spaan, 1990)] as well as its geometry (Choy and Kassab, 2009) can, in turn, alter and redistribute myocardial demand and supply in heart diseases. These confounding changes, however, seldom occur in isolation in heart diseases. As such, it is unclear how physiological and pathological changes in LV mechanics and geometry can impact both the transmural distribution of coronary flow and the myocardial work density across the LV wall to tip the myocardial demand-supply balance. This question is difficult to answer from experimental or clinical studies as it is challenging to measure myocardial blood flow *in vivo* in the deep layers of the heart wall, and it is currently not possible to measure myofiber stress and regional work density in the deep layers of the heart wall (Huisman et al., 1980).

Computational models have been developed to address the limitations associated with experiments. The integrated 3D LV (Regazzoni et al., 2020) and biventricular (Piersanti et al., 2021) electromechanical models have been proposed and coupled with a closed-loop 0D lumped parameter model describing blood circulation in the whole cardiovascular network. In another study (Augustin et al., 2020), the 3D biventricular electromechanical model has been coupled to the 0D CircAdapt closed-loop circulation model. Finite element (FE) models describing cardiac electromechanics have also been developed to investigate regional myocardial work density (Kerckhoffs et al., 2005) and its regional distribution in the LV endocardium (Gsell et al., 2018). The transmural distribution of myocardial work density and coronary perfusion across the wall of the entire heart, however, has not been investigated in these computational models. On the other hand, computational models have been developed and applied to investigate various aspects of coronary hemodynamics [e.g., the effects of myocardial contraction on coronary flow waveforms (Gregg and Sabiston, 1957), pressure wave propagation in epicardial coronary arteries (Davidson and Giraud, 2012), interaction between coronary and systemic hemodynamics (Smith, 2004), and transmural variation of coronary flow (Mynard and Smolich, 2016)]. These models, however, are focused exclusively on the coronary arteries, and do not explicitly consider the interactions between coronary perfusion, cardiac mechanics, and systemic hemodynamics. The effects of these interactions on transmural distribution of myocardial flow are also largely neglected in these studies. Recently, our group has developed computational models that strongly couples ventricular mechanics with coronary perfusion (Fan et al., 2020, 2021; Namani et al., 2020), but either the LV is highly idealized based on a time-varying elastance function (Fan et al., 2020, 2021), or an open-loop coupling between LV and perfusion is assumed (Namani et al., 2020). As such, the effects of LV mechanics, function, and geometry on myocardial demand/supply have not been comprehensively evaluated in these models.

Here, we describe a novel computational modeling framework that couples an FE model of the LV with a coronary flow analysis in a closed-loop system to investigate the transmural myocardial supply/demand across the ventricular wall. Coronary flow is analyzed under passive condition, where the intrinsic passive mechanical behavior of the vessel is considered without flow regulation. The model predictions are validated against measurements of both LV mechanics and coronary perfusion. The model is then applied to investigate how transmural distribution of myocardial blood flow and work density are affected by changes in contractility, preload, afterload, and geometry of the LV. We also introduce a novel myocardial work density-perfusion mismatch ratio to quantify changes in myocardial demand/supply balance (or imbalance) across the myocardial wall due to alterations of loading conditions, LV geometry and function. Evaluating this mismatch ratio using computational modeling helps overcome limitations associated with existing experimental metrics of myocardial demand/supply ratio.

## Materials and Methods

### Systemic Circulation Model

**FIGURE 1:**
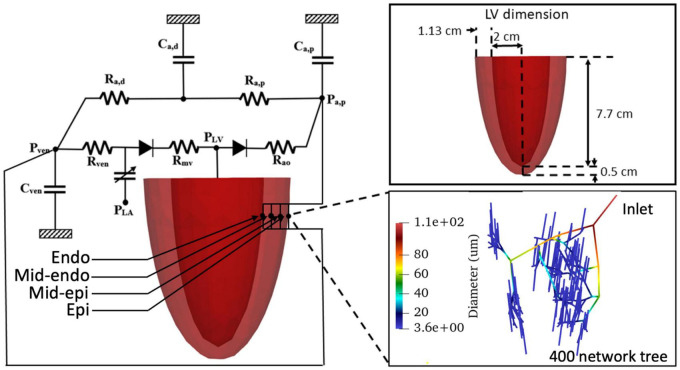
Schematic of the cardiac-coronary computational framework. The LV FE model (with dimension given in the inset) is connected to four coronary networks located at different transmural location across the myocardial wall. Each network consists of 400 vessels with different vessel radius (see inset).

The computational framework consists of an FE model of the LV and 4 coronary microvascular networks (each consisting of 400 vessels) located at different transmural locations across the LV wall (i.e., endocardium, mid-endocardium, mid-epicardium, and epicardium) that are all connected to a lumped parameter representation of the systemic circulation (Figure 1). We note that the circulatory modeling framework is an extension of a previous study of only LV mechanics (Shavik et al., 2017, 2021), in which an additional distal arterial compartment that is parameterized by a resistance *R*_*a,d*_ and a compliance *C*_*a,d*_ is included here.

The governing system of equations for the lumped parameter representation are given as follows


(1) $$\frac{dV_{LA}\left(t\right)}{dt}=q_{ven}\left(t\right)-q_{mv}\left(t\right),$$

(2) $$\frac{dV_{LV}\left(t\right)}{dt}=q_{mv}\left(t\right)-q_{ao}\left(t\right),$$

(3) $$\frac{dV_{a,p}\left(t\right)}{dt}=q_{ao}\left(t\right)-q_{a,p}\left(t\right)-\sum_{i=1}^{4}q_{cor,i}\left(t\right),$$

(4) $$\frac{dV_{a,d}\left(t\right)}{dt}=q_{a,p}\left(t\right)-q_{a,d}\left(t\right),$$

(5) $$\frac{dV_{ven}\left(t\right)}{dt}=q_{a,d}\left(t\right)-q_{ven}\left(t\right)+\sum_{i=1}^{4}q_{cor,i}\left(t\right).$$

In Eqs 1–5, *V_LA_*, *V*_*LV*_, *V*_*a*,*p*_, *V*_*a*,*d*_, and *V*_*ven*_ are the volumes of the five compartments with the subscripts denoting the left atrium (LA), LV, proximal and distal peripheral arteries, and peripheral veins, respectively. In Eqs 3, 5, *q*_*cor*,*i*_ is the flow rate associated with coronary network at transmural location *i*. We note the flow across the heart valves is unidirectional (assuming no valvular regurgitation) (Shavik et al., 2019). Flow rates in the veins, mitral and aortic valves, proximal peripheral arteries, and distal peripheral arteries are denoted by *q_ven_*, *q*_*mv*_, *q*_*ao*_,*q*_*a*,*p*_, and *q*_*a,d*_, which are determined by each segment’s resistance and the pressure difference between the two connecting storage compartments as


(6) $$q_{ao}\left(t\right)=\left\{ \begin{array}{c} \frac{P_{LV}\left(t\right)-P_{a,p}\left(t\right)}{R_{ao}}P_{LV},\left(t\right)=P_{a,p}\left(t\right)\\ 0\;P_{LV},\left(t\right)<P_{a,p}\left(t\right) \end{array}\right.,$$

(7) $$q_{a,p}\left(t\right)=\frac{P_{a,p}\left(t\right)-P_{a,d}\left(t\right)}{R_{a,p}},$$

(8) $$q_{a,d}\left(t\right)=\frac{P_{a,d}\left(t\right)-P_{ven}\left(t\right)}{R_{a,d}},$$

(9) $$q_{ven}\left(t\right)=\frac{P_{ven}\left(t\right)-P_{LA}\left(t\right)}{R_{ven}},$$

(10) $$q_{mv}\left(t\right)=\left\{ \begin{array}{c} \frac{P_{LA}\left(t\right)-P_{LV}\left(t\right)}{R_{mv}}P_{LA},\left(t\right)=P_{LV}\left(t\right)\\ 0\;P_{LA},\left(t\right)<P_{LV}\left(t\right) \end{array}\right..$$

In Eqs. 6–10, *P*_*LV*_, *P*_*a*,*p*_, *P*_*a*,*d*_, *P*_*ven*_, and *P*_*LA*_ are the cavity pressures, and *R*_*ao*_, *R*_*a,p*_, *R*_*a*,*d*_, *R*_*ven*_, and *R*_*mv*_ are the resistance of the aorta, peripheral proximal and distal arteries, peripheral veins, and mitral valve, respectively. Pressures in the storage compartments representing the peripheral (proximal and distal) arterial and venous networks are given as


(11) $$P_{a,p}=\frac{V_{a,p}\left(t\right)-V_{a,p0}}{C_{a,p}},$$

(12) $$P_{a,d}=\frac{V_{a,d}\left(t\right)-V_{a,d0}}{C_{a,d}}.$$

(13) $$P_{ven}=\frac{V_{ven}\left(t\right)-V_{ven0}}{C_{ven}},$$

where(*V*_*a*,*p*0_, *V*_*a*,*d*0_, *V*_*ven*0_) are the prescribed (arteries and veins) resting volumes, and (*C*_*a,p*_, *C*_*a*,*d*_, *C*_*ven*_) are the prescribed total (arteries and veins) compliances.

The pumping characteristics of the LA is represented by a time-varying elastance model. The LA’s instantaneous pressure, *P*_*LA*_, is related to the instantaneous volume, *V*_*LA*_, by a time-varying elastance function, *e*_*LA*_(*t*) given as follows


(14) $$P_{LA}\left(V_{LA}\left(t\right),t\right)=e_{LA}\left(t\right)P_{es}\left(V_{LA}\left(t\right)\right)+\left(1-e_{LA}\left(t\right)\right)P_{ed}\left(V_{LA}\left(t\right)\right),$$

where


(15) $$P_{es}\left(V_{LA}\left(t\right)\right)=E_{es,LA}\left(V_{LA}\left(t\right)-V_{LA0}\right),$$

(16) $$P_{ed}\left(V_{LA}\left(t\right)\right)=A_{LA}\left\{exp\left[B_{LA}\left(V_{LA}\left(t\right)-V_{LA0}\right)\right]-1\right\},$$

(17) $$e_{LA}\left(t\right)=\left\{ \begin{array}{c} \frac{1}{2}\left[\left(sin\frac{\pi }{T_{max,LA}}t-\frac{\pi }{2}\right)1\right],t=\frac{3}{2}T_{max,LA}\\ \frac{1}{2}exp\left[-\left(t-\frac{3T_{max,LA}}{2}\frac{1}{\tau }\right)\right],t=\frac{3}{2}T_{max,LA} \end{array}\right..$$

In Eq. 14, *P*_*es*_ and *P*_*ed*_ are the end-systolic and end-diastolic pressures, respectively. *E*_*es*,*LA*_ is the maximal chamber elastance of LA, *V*_*LA*0_ is the volume at zero end-systolic pressure, and both *A*_*LA*_ and *B*_*LA*_ are parameters defining the end-diastolic pressure volume relationship of the LA. In Eq. 17, *T*_*max,LA*_ is the time to end systole, and τ_*LA*_ is the relaxation time constant.

### Left Ventricular Finite Element Model

Finite element formulations of the LV model were described earlier (Arumugam et al., 2019; Shavik et al., 2020; Mojumder et al., 2021). Details of the formulations are given in Supplementary Appendix C. Briefly, a quasi-static active stress formulation is used to describe the mechanical behavior of the LV. The functional relationship between pressure and volume in the LV (i.e., *P*_*LV*_ = *f*(*V_LV_*, *t*) that can connect between Eqs. 2, 10 in the lumped parameter representation of the systemic circulation) is obtained by minimizing a Lagrangian function consisting of myocardial tissue strain energy function and terms associated with the enforcement of constraints on **(1)** myocardial tissue incompressibility, **(2)** zero-mean rigid body translation and rotation, and **(3)** cavity volume. Additionally, the LV base is constrained from moving out of the plane. Refer Arumugam et al. (2019) for details. The mechanical behavior of the LV is modeled using an active stress formulation, in which the passive mechanical behavior is described by a Fung-type strain energy function (Guccione et al., 1995) and the active mechanical behavior is described by a modified time-varying elastance model with the active second Piola-Kirchhoff (PK2) stress tensor given as


(18) $${\text{S}}_{\text{LV},a}=T_{max}\frac{Ca_{0}^{2}}{Ca_{0}^{2}ECa_{50}^{2}}C\left(t\right){\text{e}}_{f_{0}}⊗{\text{e}}_{f_{0}},$$

where **e**_*f*_0__ is the local vector defining the muscle fiber direction in the reference configuration, *T*_*max*_ is the isometric tension achieved at the longest sarcomere length, and *Ca*_0_denotes the peak intracellular calcium concentration. We also refer *T*_*max*_ as the myocardial contractility. The length dependent calcium sensitivity *ECa*_50_ and the time elastance function *C(t)* are given by


(19) $$ECa_{50}=\frac{{\left(Ca_{0}\right)}_{max}}{\sqrt{\exp\left(B\left(l-l_{0}\right)\right)-1}}$$

(20) $$C\left(t\right)=\left\{ \begin{array}{c} \frac{1}{2}\left(1-\cos\left(\frac{\pi t}{t_{0}}\right)\right),t=T_{trans}\\ \frac{1}{2}\left(1-\cos\left(\frac{\pi T_{trans}}{t_{0}}\right)\right)\text{exp}\left[-\left(\frac{t-T_{trans}}{\tau }\right)\right],t=T_{trans} \end{array}\right.,$$

where *B* is a constant, (*Ca*_0_)_*max*_ is the maximum peak intracellular calcium concentration, *l*_0_ is the sarcomere length at which no active tension develops, *l* is sarcomere length obtained from the myofiber stretch, *t*_0_ is the time taken to reach peak tension, τ is the relaxation time constant, and *T*_*trans*_ is the transition time. We note that the time-varying elastance function prescribed in Eq. 20 is slightly different from the original one prescribed in the Guccione model (Guccione et al., 1993), but is analogous to that prescribed for the LA in Eq. 17. We have made this modification as it enables us to control the relaxation time constant τ of the pressure waveform more directly, while retaining the peak force–sarcomere length relationship of the cardiac muscle. The FE model of the LV was implemented using FEniCS (Logg et al., 2012), an open-source FE library.

### Coronary Flow Analysis

A coronary microvascular network consisting of 400 vessels is used to represent the coronary microcirculation at each of the 4 transmural locations across the LV wall. The 400 network was pruned from a previously reconstructed coronary network (Namani et al., 2018), consisting 195 bifurcations, 3 trifurcations, and 79 terminal vessels. The microvascular tree that is an input to the flow analysis, was generated from the global statistics of the measured coronary vessel diameters (Kassab et al., 1993). Collateral flow between the networks at different transmural locations is not considered here since they are functional in only about 1/5 of humans (Marchi, 2014). Briefly, flow in each vessel of the coronary arterial microvascular network under passive condition, where the intrinsic passive mechanical behavior of the vessel is considered without flow regulation, is modeled by a non-linear three-element Windkessel electrical representation. Defined subsequently in the text as passive coronary flow, this quantity is comparable to coronary flow measured under maximally vasodilated condition that can be achieved with the administration of adenosine to the vessels in experiments. Applying mass conservation at the Windkessel’s internal (”mid”) node in each single vessel *i* results in the following ordinary differential equation (ODE),


(21) $$\frac{P_{in}^{i}-P_{mid}^{i}}{R_{1}^{i}}+\frac{P_{out}^{i}-P_{mid}^{i}}{R_{2}^{i}}+\frac{d}{dt}\left[C^{i}\left(P_{T}^{i}-P_{mid}^{i}\right)\right]=0,i=1,2,3,,n,$$

where $P_{T}^{i}$ is the IMP imposed on the vessel and *n* is the number of vessels. For each of the four networks (Figure 1), we prescribed a homogeneous $P_{T}^{i}$ using the local value of the Lagrange multiplier associated with the incompressibility constraint from the FE LV model (Arumugam et al., 2019; Shavik et al., 2020). We note that the Lagrange multiplier is associated with the hydrostatic pressure of the fluid-like ground matrix in constrained mixture models of the biological tissue (Huyghe et al., 2004; Hollander et al., 2011), and hence, is assumed here to be the extravascular pressure imposed on the coronary micro-vessels. As the distance between each vessel’s location is very small relative to the LV dimension, IMP ($P_{T}^{i}$) is assumed to be uniform in each vessel within a network. We note, however, that IMP ($P_{T}^{i}$) is different in the networks that are located at four different transmural locations. In Eq. 21, the resistances *R*_1_ and *R*_2_ are assumed to be the same and are given as


(22) $$R_{1}=R_{2}=\frac{64\mu \cdot L}{\pi \cdot D{\left(t\right)}^{4}},$$

whereas the capacitance *C* is given by


(23) $$C=\frac{\partial \left(\pi \cdot D{\left(t\right)}^{2}L/4\right)}{\partial \left(P_{mid}\left(t\right)-P_{T}\left(t\right)\right)}.$$

**FIGURE 2:**
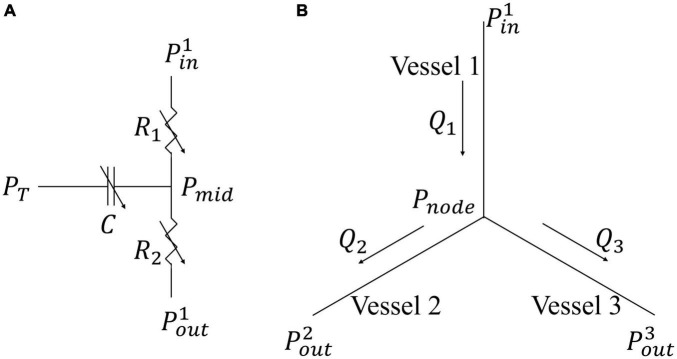
Flow models in a single vessel and vessel bifurcation. **(A)** Electrical non-linear analog of the segment lumped model; **(B)** Single bifurcation three-vessel network.

In Eq. 22, *L* and *D*(*t*) are the length and diameter of a single vessel, and μ is a blood viscosity, which is assumed to be a constant 2.7×10^−9^ MPa⋅s to simplify the flow analysis (Pries et al., 1994; Figure 2A). The vessel diameter is prescribed as


(24) $$D\left(t\right)=2B_{p}+\frac{2\left(A_{p}-B_{p}\right)}{\pi }\left[\frac{\pi }{2}arctan\left(\frac{P-\phi_{p}}{C_{p}}\right)\right],$$

where *A_p_* and *B*_*p*_ are the asymptotical highest and lowest radii, respectively (under the highest and lowest time-dependent transvascular pressure, *P*), ϕ_*p*_ is the transvascular pressure corresponding to the mean of radii *A_p_* and *B_p_*, and *C_p_* is the passive response bandwidth. The transvascular pressure *P* is determined by the pressure difference between mid-node and the local IMP. Passive flow in a single vessel is governed by the Poiseuille relation as


(25) $$Q\left(t\right)=\frac{\pi \cdot \frac{D{\left(t\right)}^{4}}{16}\left(P_{in}\left(t\right)-P_{out}\left(t\right)\right)}{8\mu \cdot L},$$

where *Q*(*t*) is the flow rate and (*P*_*in*_(*t*)−*P*_*out*_(*t*)) is the pressure difference between the vessel’s inlet and outlet. Mass conservation is satisfied in the coronary network at each nodal position connecting the vessels, namely,


(26) $$\sum_{i=1}^{j}Q_{i}\left(t\right)=0,$$

where *Q_i_* is the flow rate in each vessel and *j* is the number of vessels connected to the node (e.g., *j* = 3 for a bifurcation and *j* = 4 for a trifurcation) (Figure 2B). As the resistance and capacitance are not constants, the resultant system of ODEs with $$$*P^i*_*mid*_ of the vessels as the unknowns is non-linear. The system of ODEs is solved using the backward differentiation formula method in CVODE^1^ with pressure at the network’s inlet and outlets prescribed as boundary conditions. The parameters used for the flow analysis in Eqs 21–26 are similar to previous work (Namani et al., 2018; Fan et al., 2020). These parameters are tabulated in Supplementary Table B4.

An explicit time-integration scheme is used for the ODEs. Specifically, pressures in all compartments calculated from Eqs 11–14 based on the blood volumes and prescribed resting volumes (LV pressure based on FE model) can be substituted into Eqs 6–10 for computing flow rates. Then, blood volume at current time step can be obtained by these flow rates with prescribed time step and blood volume in previous time step based on Eqs 1–5. The explicit time-integration scheme is easier to implement and less time-consuming as compared to the implicit scheme. Since explicit time-integration scheme is not unconditionally stable, we have chosen a smaller time step that is sufficient for numerical stability. The LV and coronary models are coupled bidirectionally with the lumped parameter representation of the systemic circulation at each time step as described below. ***First*,** LV volume computed from Eq. 2 in the systemic circulation is applied to compute the LV pressure in the FE model (Supplementary Eq. C4), which in turn is fed into Eqs 6, 10 in the lumped parameter model to couple the LV and systemic circulation. ***Second***, the proximal arterial pressure (*P*_*a*,*p*_ in Eq. 11) and venous pressure (*P*_*ven*_ in Eq. 13) in the systemic circulation serve as boundary conditions in each coronary network to compute for the total coronary flow at different transmural locations. Total inlet and outlet coronary flow computed with these pressure boundary conditions are fed back into the lumped parameter model in Eqs 3, 5 to couple the coronary and systemic circulation. ***Third*,** IMP in the coronary models is prescribed using the local value of the Lagrange multiplier associated with the incompressibility constraint of the FE LV model (from solving Supplementary Eq. C4) to couple the LV and coronary models. The rationale for prescribing the local Lagrange multiplier as IMP is given in the section “Discussion.”

### Simulation Cases

We first calibrate the computational framework to obtain the waveforms of LV pressure, volume, and strains that are in agreement with those measured in a typical normal human LV or in a large animal under normal conditions. Specifically, model parameters were chosen and ***calibrated*** so that LV pressure, volume waveforms as well as circumferential and longitudinal strain waveforms are in agreement with measurements in healthy humans (Gorcsan and Tanaka, 2011; Hoit, 2011; Smiseth et al., 2016; Shavik et al., 2019). In the lumped model, the parameters are selected based on previous work (Shavik et al., 2017), which are then manually adjusted to match the data. Specifically, the arterial compliance (*C*_*art*_), the peripheral arterial resistance (*R*_*per*_), the arterial resting volume (*V*_*art*0_), and the initial value for the arterial volume (*V*_*art*_) are varied to adjust both the peak and the end systolic arterial pressures. The LV volume is adjusted by varying the initial value of the LV volume (*V*_*LV*_) before simulation of the cardiac cycle begins, and the venous compartment’s resting volume (*V*_*ven*0_). Parameters used in the FE model of the LV and coronary perfusion are from previous works Guccione et al. (1995) and Fan et al. (2020), respectively.

Model predictions of the transmural distributions of IMP/LV pressure ratio, normalized passive coronary flow and myocardial work density are, respectively, ***validated*** (i.e., without any other adjustment of parameters) against IMP (Heineman and Grayson, 1985) and flow measurements (under full vasodilation) (Vatner and Hittinger, 1993) from normal canine model. Because local myocardial work density is not directly measurable in experiments, model prediction of its transmural distribution is ***corroborated*** with quantities indicative of the myocardial work density, namely, **(1)** MVO_2_ or cardiac metabolism, **(2)** regulated coronary flow, and **(3)** myocyte cell size (see section “Discussion” for more details).

After model calibration, we then vary the parameters to investigate the independent effects of either contractility, preload, afterload, or geometry (cavity volume and wall thickness) on the LV pressure, arterial pressure, transmural distribution of IMP, coronary flow, and myocardial work density. The range of each parameter is selected to produce physiological and pathophysiological conditions in heart diseases. Specifically, it has been measured that myocardial contractility assessed by the peak gradient of the LV pressure can be altered by 50% in diseases or by inotropic drugs (Pouleur et al., 1982) whereas contractility represented by *E*_*es*_ in heart failure with preserved ejection fraction patients is about twice of that in control cases (Kawaguchi et al., 2003). Peak LV pressure can be increased to around 160 mmHg by enhanced preload or afterload under abnormal conditions, e.g., hypertension (Lavine and Lavine, 2006), or during exercise (Ehsani et al., 1986). End-diastolic pressure can vary between 4 and 12 mmHg under normal conditions (Peverill, 2020), and increase up to around 25 mmHg in disease conditions (Mielniczuk et al., 2007). Left ventricular wall thickness can vary between 0.43 (Kawel et al., 2012) and 3 cm in patients with cardiac hypertrophy (Olivotto et al., 2003; Kawel et al., 2012). End-diastolic volume is as high as 200 ml in patients with eccentric hypertrophy (Lee et al., 2012) and is as low as 60 ml in patients with mitral valve diseases (McDonald, 1976). Correspondingly, we vary each parameter over a range of values in the model by decreasing and increasing the calibrated baseline value. The specific model parameters and their values are given as follows to reproduce these clinical conditions.

(1) Myocardial contractility with *T*_*max*_ varying between 65 and 205 kPa;
(2) Afterload (indexed by peak LV pressure) with resistance of peripheral distal arteries *R*_*a,d*_ varying between 33 and 318 kPa⋅ms/ml and resting volume of the veins *V*_*ven*0_ adjusted to keep the preload (indexed by end-diastolic pressure) constant at 8 mmHg;
(3) Preload (indexed by end-diastolic pressure, EDP) by varying *V*_*ven*0_ between 3707 and 2696 ml;
(4) Wall thickness varying between 0.53 and 1.73 cm at constant afterload and preload by adjusting *R*_*a,d*_ and *R*_*per*_;
(5) LV cavity (unloaded) volume varying between 65 and 205 ml at constant afterload and preload by adjusting *R*_*a,d*_ and *R*_*per*_.

We note that instead of perturbing individual model parameters by a fixed percentage, the sensitivity analysis was performed based on isolated changes of ventricular loading (preload and afterload), LV intrinsic function (contractility), and geometry over the range encountered under different physiological or pathological conditions as described above. This approach helps to ensure that the relative contributions of model parameters are physiologically meaningful. We further note that the wall thickness was varied without considering any changes in the coronary network, which will result in a change in capillary density.

Mesh size sensitivity studies were performed to determine the number of elements required for the transmural distribution of myofiber stress, strain and work density, as well as global quantities (e.g., pressure and volume waveforms) to converge. The number of elements was varied between 1159 and 54773, and it was found that the solution is converged at between 4353 and 54773 elements, which is comparable with that found in a previous study (Lim et al., 2015; Supplementary Appendix A1). The simulation is ran in Michigan State University High Performance Computing Cluster with 8 processors with each having a base clock speed of 2.5 GHz. The time step is set as 1 ms in the cardiac cycle and the simulation is terminated when the results reached a steady periodic state, usually within 6 cycles in less than 4 h with 4353 elements.

### Post-processing of Results

Results from the computational framework were obtained from each simulation case. Following Kerckhoffs et al. (2005) and Walmsley et al. (2015), local work density in a cardiac cycle is given by the area of the myofiber stress-strain loop i.e.,


(27) $$W_{f}=\int_{cardiaccycle}S_{ff}.dE_{ff},$$

where *S_ff_* and *E*_*ff*_ are PK2 fiber stress and Green-Lagrange fiber strain, respectively. Because active stress is prescribed to develop only in the myofiber direction, we have considered only work in that direction, and have neglected work associated with other components of the stress and strain tensor as considered in other studies (Gsell et al., 2018). The local Green-Lagrange fiber strain is defined as


(28) $$E_{ff}=\frac{1}{2}\left({\text{e}}_{f}\cdot \text{C}\cdot {\text{e}}_{f}-1\right),$$

where **C** is the right Cauchy-Green deformation tensor and **e_f_** is the unit vector in the myofiber direction in the reference configuration. We note that *W*_*f*_ represents only the local mechanical work density and does not consider basal metabolism and other chemical energies. The local myofiber stress-strain loop area represents the net work performed by cells locally in the tissue (Wang et al., 2012). As such, there is a basis for using area of the myofiber stress-strain loop as an index for local work density performed by the cell.

Transmural distributions of *W*_*f*_ and IMPwere obtained based on their value at around 10,000 randomly distributed locations across the LV wall together with their corresponding radial position *R*, with *R* = 0 denoting the endocardium and *R* = 1 denoting the epicardium. The number of points was determined to be sufficient for convergence (i.e., the distributions do not change with the inclusion of more points). Normal (Euler-Almansi) strains in the circumferential (*e*_*cc*_)and longitudinal (*e*_*ll*_) directions were also computed with end-diastole serving as the reference configuration (Shavik et al., 2017) by


(29) $$e_{ii}=\frac{1}{2}\left(1-\frac{1}{{\text{e}}_{i}\cdot \text{C}\cdot {\text{e}}_{i}}\right),i=candl.$$

In the above equation, **e_c_** and **e_l_** denote the circumferential and longitudinal directions, respectively. Total passive coronary flow from each coronary microvascular network at different transmural locations was computed by integrating the flow rate *q*_*cor*,*i*_(*t*) over the cardiac cycle.

## Results

### Baseline Case

**FIGURE 3:**
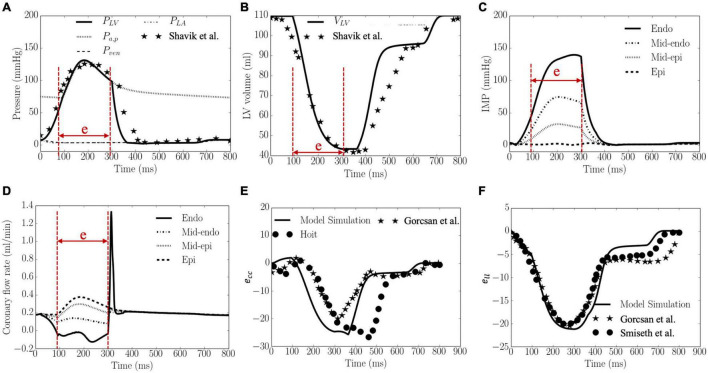
Model predicted results. **(A)** Pressures, **(B)** LV volume, **(C)** IMP, **(D)** Coronary flow rate, **(E)** *e*_*cc*_, and **(F)** *e*_*ll*_ waveforms. LV pressure and volume, and *e*_*cc*_ and *e_ll_* are plotted together with measurements from 3D echocardiography (dots and stars), and were in agreement with clinical measurements in Gorcsan and Tanaka (2011), Hoit (2011) and Smiseth et al. (2016), Shavik et al. (2019), respectively. “e” in panels **(A–D)** indicates “ejection phase.”

Waveforms of the pressures in the LV and artery, LV volume, IMP, *q*_*cor*,*i*_, *E*_*cc*_ and *E*_*ll*_ are shown in Figure 3. The calibrated model parameters are tabulated in Tables B1–3 in Supplementary Appendix B. Left ventricular ejection fraction (EF), end-diastolic volume (EDV), end-systolic volume (ESV) were 61%, 110 and 43 ml, respectively. Peak LV pressure, LVEDP, systolic and diastolic arterial pressures were 131, 8, 130, and 73 mmHg, respectively. The LV pressure and volume waveforms matched with measurements of normal humans. Peak IMP at each transmural location where the coronary microvascular network is located, varies from 140 mmHg at the endocardium to 3 mmHg at the epicardium. Peak *E*_*cc*_ and *E*_*ll*_ were 26 and 21%, respectively, and their waveforms were in agreement with 3D echo measurements from normal humans. Intramyocardial pressure waveform was highest at the endocardium (*R* = 0) and decreased transmurally toward the epicardium (*R* = 1). Passive coronary flow rate was largely smaller during systole (“e” phase) in the inner layers of the myocardium at mid-endocardium and endocardium when compared to that during diastole. At the endocardium (*R* = 0), passive coronary flow rate was negative during systole. Passive coronary flow rate at the endocardium (*R* = 0) and mid-endocardium during systole (*t* = 0–300 ms) is dominated by the effects of IMP. At the onset of diastole in the isovolumic relaxation phase (*t* > 300 ms), passive coronary flow rate increased rapidly and produced a high peak in the waveform because IMP fell substantially faster from its peak value to the filling pressure (with a drop of more than 100 mmHg) than the reduction in arterial pressure (from 100 to 80 mmHg). The high peak arises because of the fast decay in the IMP. Slowing the decay by increasing the isovolumic relaxation time constant (indexed by τ) from 25 to 105 ms (see “Sensitivity Analysis” in Supplementary Appendix F) reduced the peak magnitude in the coronary flow rate waveform at the endocardium (Supplementary Figure F1C). Except for the high peak associated with the endocardium, coronary flow rate in the microvascular networks were largely similar during diastole.

**FIGURE 4:**
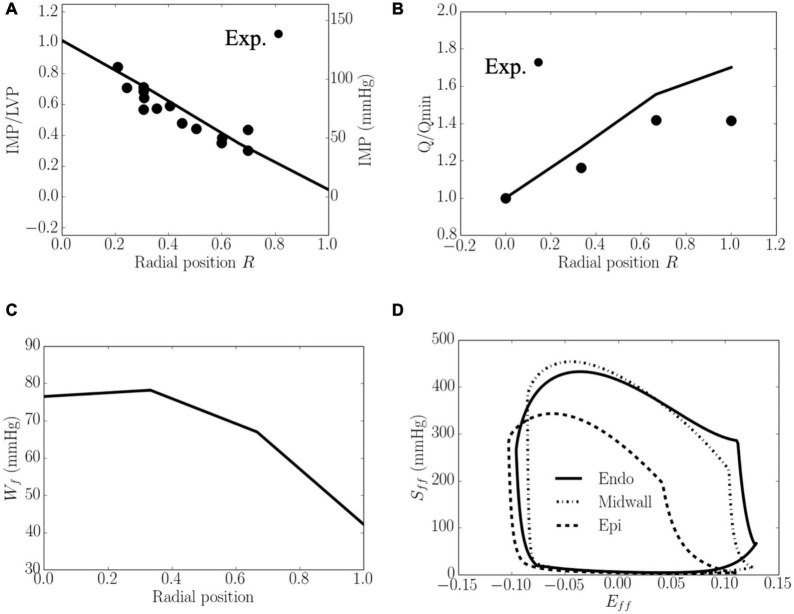
Transmural distribution. **(A)** IMP that normalized against LV pressure, is comparable to experimental measurements (Heineman and Grayson, 1985); **(B)** Total coronary flow over a cardiac cycle that is normalized by flow at the endocardium (*R* = 0), is comparable to the measurements in a canine model under maximal coronary vasodilation (Vatner and Hittinger, 1993); **(C)** Work done over a cardiac cycle *W*_*f*_.**(D)** Average *S*_*ff*_−*E*_*ff*_ loop at the endocardial (*R* = 0), mid-wall and epicardial (*R* = 1) regions. ⋅ denotes experimental measurements.

Total coronary flow increased while both *W_f_* and IMP decreased transmurally from the endocardium (*R* = 0) to the epicardium (*R* = 1) (Figure 4). The transmural variation of these quantities is approximately linear. Specifically, the mean value of peak IMP was approximately 1.0 times the LV pressure at the endocardium (*R* = 0) and decreased to around 0 at the epicardium (*R* = 1) (Figure 4A), consistent with experimental measurements. Total coronary flow was largest at the epicardium (*R* = 1) and approximately 1.7 times that at the endocardium (*R* = 0), which has the smallest total coronary flow (Figure 4B). This result is also largely consistent with experimental measurements. Total myocardial work density over a cardiac cycle *W_f_* was approximately 1.7 times higher at the endocardium (*R* = 0) than that at the epicardium (*R* = 1) (Figure 4C). The larger value of *W_f_* at the endocardial (*R* = 0) and mid-wall (*R* = 1/2) regions is the result of a higher peak myofiber stress in this region compared to that at the epicardium (*R* = 1) (Figure 4D).

### Effects of Contractility

**FIGURE 5:**
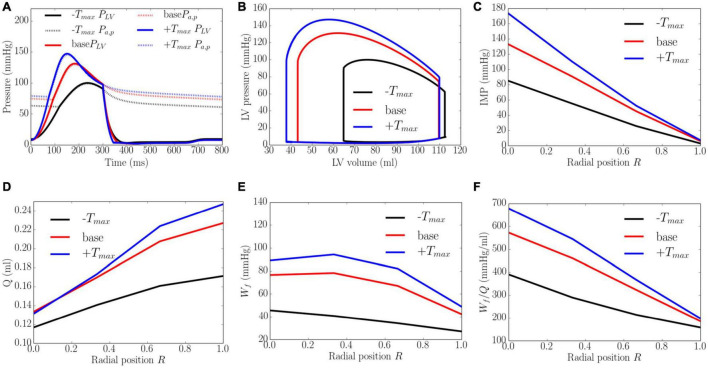
Effects of myocardial contractility on **(A)** Pressure waveforms; **(B)** LV pressure-volume loop; Transmural distribution of **(C)** IMP; **(D)** Total coronary flow over a cardiac cycle; **(E)** Work density over a cardiac cycle *W_f_*; **(F)** Work density-perfusion mismatch index ratio *W*_*f*_/*Q*. “-*T*_*max*_,” “base,” and “+*T*_*max*_″ denote *T*_*max*_ = 65, 130, and 205 kPa.

Increasing the myocardial contractility *T*_*max*_ from a minimum value of 65 kPa to a maximum value of 205 kPa led to an increase in EF from 43 to 66% and an increase in the peak LV pressure from 100 to 143 mmHg. This resulted in an increase in the transmural gradient of IMP (Figure 5). Given that the coronary flow is a compromise between perfusion pressure (that is controlled by the peak LV pressure) and IMP, increasing *T*_*max*_ from 65 to 130 kPa produced an increase in the coronary flow at endocardium (14.3%). A further increase in *T*_*max*_ from 130 to 205 kPa, however, produced a decrease in the coronary flow by 1.6% at the endocardial region (*R* = 0). In the mid-endo (*R* = 1/3), mid-epi (*R* = 2/3), and epicardial (*R* = 1) regions, an increase in *T*_*max*_ produced a monotonic increase in coronary flow. In terms of myocardial work density, the transmural gradient of *W_f_* remained relatively unchanged with increasing *T*_*max*_. Defining the ratio *W*_*f*_/*Q* as a work density-perfusion mismatch index, this quantity is substantially increased at the endocardium (*R* = 0) with increasing contractility. Specifically, the work density-perfusion mismatch was increased by ∼178% at the endocardium (*R* = 0) with a 215% increase in contractility. When *T*_*max*_ was increased from 65 to 205 kPa, the endo/epi ratio of *W*_*f*_/*Q* was increased from 2.28 to 3.21, implying a greater mismatch at the endocardium (*R* = 0).

### Effects of Afterload

**FIGURE 6:**
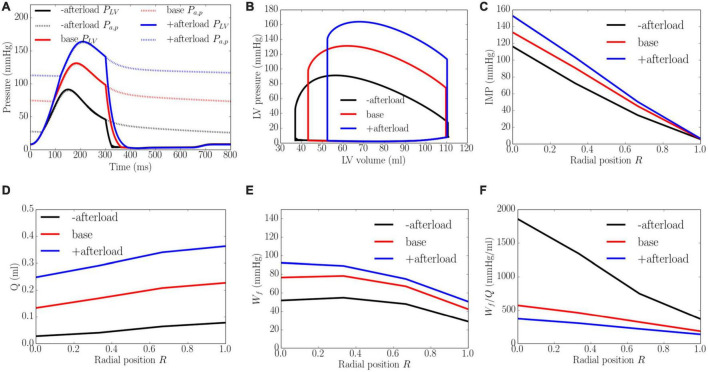
Effects of afterload on **(A)** Pressure waveforms; **(B)** LV pressure-volume loop; Transmural distribution of **(C)** IMP; **(D)** Total coronary flow over a cardiac cycle; **(E)** Work density over a cardiac cycle *W_f_*; **(F)** Work density-perfusion mismatch index ratio *W*_*f*_/*Q*. “-afterload,” “base,” and “+afterload” denote reduced afterload, baseline, and increased afterload cases.

An increase from the minimum to maximum value in the afterload at a constant preload led to a decrease in EF from 67 to 52% and an increase in peak LV pressure from 91 to 164 mmHg, which in turn, caused the transmural gradient of IMP to increase (Figure 6). An increase in afterload from the baseline produced a more significant increase in peak LV pressure (by 80.2%) than IMP (varies from 109 to 145 mmHg, 33.0% at the endocardium (*R* = 0) and from 10 to 14 mmHg, 30.0% in the epicardium, *R* = 1). Because inlet pressure also increased from an average of 35 mmHg in the lowest afterload case to 125 mmHg in the highest afterload case, coronary flow is increased monotonically in all layers of the myocardium as a result. Specifically, coronary flow increased by 8.9, 7.1, 5.3, and 4.6 times at the endocardial (*R* = 0), mid-endo (*R* = 1/3), mid-epi (*R* = 2/3) and epicardial (*R* = 1) regions. In terms of the myocardial work density, a higher afterload (cf. baseline) produced an increase in the transmural gradient of *W_f_*. The variation of *W_f_* also became more linear across the whole wall, where *W_f_* at the endocardium (*R* = 0) was 1.7 times that at the epicardium (*R* = 1). At a lower afterload (cf. baseline), *W_f_* was approximately homogeneous from endocardium (*R* = 0) to the mid-wall before reducing linearly with transmural location. Myocardial work density at the endocardium (*R* = 0) was 1.6 times that at the epicardium (*R* = 1). As there is a larger percentage increase in total coronary flow than that of myocardial work density across the myocardial wall, the work density-perfusion mismatch *W*_*f*_/*Q* was reduced with increasing afterload, with the largest increase occurring at the endocardium (*R* = 0).

### Effects of Preload

**FIGURE 7:**
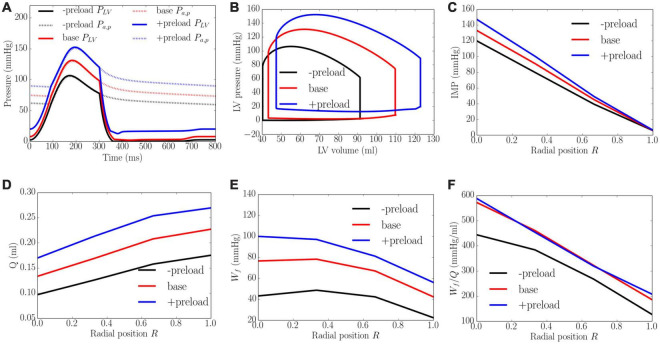
Effects of preload on **(A)** Pressure waveforms; **(B)** LV pressure-volume loop; **(C)** Transmural distribution of IMP; **(D)** Total coronary flow over a cardiac cycle; **(E)** Work density over a cardiac cycle *W_f_*; **(F)** Work density-perfusion mismatch index ratio *W*_*f*_/*Q*. “-preload,” “base,” and “+preload” denote reduced preload, baseline, and increased preload cases.

Increasing preload from the minimum to the maximum value led to an increase in the peak LV pressure, EDP, and diastolic blood pressure (DBP) from 106 to 152 mmHg, 2 to 20 mmHg, and 59 to 88 mmHg, respectively. It also increased the EF slightly from 56 to 61% (Figure 7). An increase in preload produced an increase in IMP from 125 to 155 mmHg at the endocardium (*R* = 0) but IMP remained close to 0 at the epicardium (*R* = 1). Coronary flow in all layers of the myocardium was increased with increasing preload, with the largest increase of 1.8 times occurring at the endocardium (*R* = 0) and the least increase of 1.5 times occurring at the epicardium (*R* = 1). An increase of preload also produced an increase in *W*_*f*_ across the wall as well as its transmural gradient. At the highest preload, *W_f_* was 2.3 times of that at the lowest preload at both the endocardium (*R* = 0) and epicardium (*R* = 1). Unlike the increase in afterload, the increase in total coronary flow was less than the increase in myocardial work density, which causes the work density-perfusion mismatch *W*_*f*_/*Q* to increase with increasing preload in all layers of the myocardium (1.3 times at the endocardium, *R* = 0 and 1.5 times at the epicardium, *R* = 1).

### Effects of Wall Thickness

**FIGURE 8:**
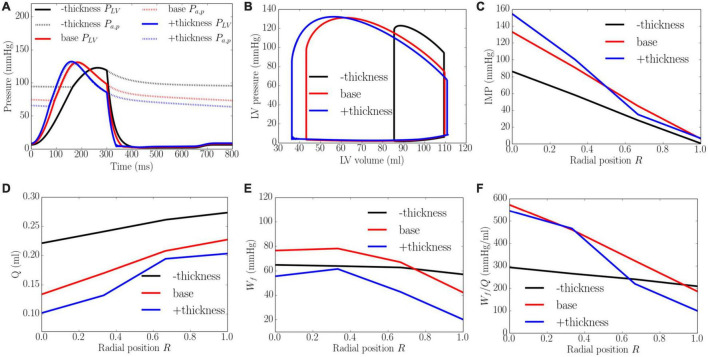
Effects of wall thickness on **(A)** Pressure waveforms; **(B)** LV pressure-volume loop; Transmural distribution of **(C)** IMP; **(D)** Total coronary flow over a cardiac cycle; **(E)** Work density over a cardiac cycle *W_f_*; **(F)** Work density-perfusion mismatch index ratio *W*_*f*_/*Q*. “–thickness,” “base,” and “+thickness” denote reduced thickness, baseline, and increased thickness cases.

An increase of wall thickness from the minimum to the maximum value with a constant preload (EDP ∼8 mmHg) and afterload (peak LV pressure ∼141 mmHg) produced an increase in EF from 23 to 68% (Figure 8). It also increased the transmural gradient of IMP, with a relatively larger increase occurring at the endocardium (*R* = 0) (from 97 to 165 mmHg) than at the epicardium (*R* = 1) (from 7 to 10 mmHg). Because of the increase in IMP, total coronary flow was reduced in all layers across the myocardial wall. Myocardial work density *W_f_*, on the other hand, varies non-monotonically with changes in the wall thickness. At a smaller wall thickness (cf. baseline), *W_f_* was approximately homogeneous across from endocardium (*R* = 0) to epicardium (*R* = 1). Although strain was reduced compared to the baseline case, stress was increased and as a result, myocardial work density was constant across the wall (Figure E1 in Supplementary Appendix E). When wall thickness was increased (cf. baseline), the transmural variation of *W_f_* is similar to that at baseline. Myocardial work density, however, was reduced across the wall. As a result, the work density-perfusion mismatch *W*_*f*_/*Q* varied differently across the myocardial wall when wall thickness was increased. When wall thickness increased from 0.53 to 1.73 cm, *W*_*f*_/*Q* was increased largely at the endocardium (*R* = 0) but reduced at the epicardium (*R* = 1). The endo/epi ratio of *W*_*f*_/*Q* was increased from 1.38 to 4.87, implying a more significant mismatch at the endocardium (*R* = 0) with increasing wall thickness.

### Effects of Cavity Volume

**FIGURE 9:**
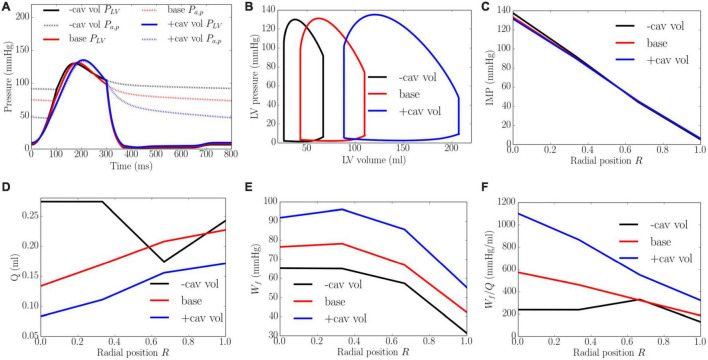
Effects of cavity volume on **(A)** Pressure waveforms; **(B)** LV pressure-volume loop; Transmural distribution of **(C)** IMP; **(D)** Total coronary flow over a cardiac cycle; **(E)** Work density over a cardiac cycle *W_f_*; **(F)** Work density-perfusion mismatch index ratio *W*_*f*_/*Q*. “-cav vol,” “base,” and “+cav vol” denote reduced cavity volume, baseline, and increased cavity volume cases.

An increase of LV cavity volume from the minimum to the maximum value with a constant preload (EDP ∼8 mmHg) and afterload (peak LV pressure ∼138 mmHg) produced a decrease in EF from 64 to 58% and diastolic blood pressure (Figure 9). Transmural distribution of IMP was relatively unchanged with increasing cavity volume (as LV pressure was relatively unchanged). Increasing cavity volume, however, caused coronary flow to be largely reduced across the transmural wall, except in the epi-mid region where flow first increased and then decreased. Myocardial work density *W_f_* was increased with increasing cavity volume but its transmural variation did not change. As a result of the decrease in total coronary flow and increased myocardial work density, the work density-perfusion mismatch *W*_*f*_/*Q* increased with increasing cavity volume across all the myocardial wall, with the largest increase occurring at the endocardium (*R* = 0). The endo/epi ratio of *W*_*f*_/*Q* was increased from 1.65 to 3.26, implying a more significant mismatch with increasing cavity volume.

## Discussion

The overall finding of this study is that LV contractility, afterload, preload and geometry (wall thickness and cavity volume) affect both the transmural distribution of coronary flow and myocardial work density, which in turn, affect the transmural work density-perfusion mismatch in the heart wall. The specific key findings are: **(1)** Transmural distribution of IMP across the LV wall can explain the transmural distribution of passive coronary flow (varying monotonically from endocardium, *R* = 0 to epicardium, *R* = 1) measured experimentally (Heineman and Grayson, 1985; Krams et al., 1989b; Vatner and Hittinger, 1993); **(2)** Coronary flow (under passive condition) *Q* at all transmural positions across the myocardial wall increases with either increasing LV contractility, afterload or preload, while it decreases with increasing wall thickness or cavity volume (at same preload and afterload); **(3)** Myocardial work density *W*_*f*_ at all transmural positions increases with either increasing LV contractility, afterload, preload or cavity volume, but reduces with increasing wall thickness; **(4)** Work density-perfusion mismatch (*W*_*f*_/*Q*) increases (worsened) with either increasing LV contractility, preload, wall thickness or cavity volume, with the increase largely occurring at the endocardium. With increasing afterload, however, the mismatch is reduced with the reduction largely occurring at the endocardium.

To the best of our knowledge, this is the first systematic computational study that describes the LV demand-supply coupling and investigates the effects of myocardial contractility, loading conditions (afterload and preload), and ventricular geometry (LV cavity volume and wall thickness) on the transmural distribution of coronary perfusion and myocardial work density. To provide insights into the effects of these parameters on myocardial demand/supply relationship, we also introduced and evaluated the transmural variation of a novel myocardial work density-perfusion mismatch ratio across the myocardium. As discussed later in detail, while myocardial supply is a real quantity that can be measured experimentally, myocardial demand is, in principle, a “virtual parameter” that cannot be measured (Heusch, 2019). Only myocardial oxygen consumption can be measured, which is indicative of the myocardial demand only if there is adequate coronary reserve to fulfill that demand (and not when the reserve is exhausted). This calls into question if existing experimentally measured indices are truly describing myocardial demand/supply mismatch. We addressed this issue here with a work density-perfusion mismatch ratio predicted by the model to gain insights into the conditions that alter myocardial demand/supply relationship. This study also overcomes the challenges in pure experimental studies, where it is difficult to isolate confounding factors as well as measuring flow, pressure and myocardial work density in the deep layers of the myocardium. To enable this *in silico* study, we have developed a cardiac-vascular modeling framework that couples the systemic circulation with an FE model of the LV and coronary perfusion in a closed-loop system. The model was **calibrated** to fit clinical measurements of the LV pressure, volume and strain waveforms (Figure 3) and its prediction of the (normalized) transmural distribution of coronary flow, IMP/LV pressure ratio and myocardial work density (Figure 4) are **validated** and **corroborated** against experimental measurements (Heineman and Grayson, 1985; Hoit, 2011; Smiseth et al., 2016; Shavik et al., 2019). As an additional validation, the calibrated model is also applied to predict coronary flow in the LV during systole under isobaric conditions (with zero cavity pressure). The prediction that coronary flow is reduced and sensitive to myocardial contractility even under isobaric conditions is also consistent with experiments (Krams et al., 1989b; Supplementary Appendix D).

### Transmural Distribution of Intramyocardial Pressure

Our model predicted that IMP decreases linearly as a function of the transmural depth from a maximum value at the endocardium (*R* = 0) to a minimum value at the epicardium (*R* = 1) in the pressurized LV (Figure 4A). The model predicted transmural variation agrees with experimental measurements (Dieudonné, 1967; Brandi and Mcgregor, 1969; Armour and Randall, 1971; Kassab et al., 2013). Specifically, the ratio of systolic peak IMP to LV pressure (IMP/LVP ratio) at the endocardium and epicardium in the baseline case compares favorably with *in vivo* micropipette measurements in dogs (1.1 at endocardium to -0.15 at the epicardium) (Heineman and Grayson, 1985). We note that the hydrostatic pressure that enforces tissue incompressibility largely (but not exactly) follows the myocardial fiber stress waveform (especially the active fiber stress) and is not directly related to the LV pressure or the cavity-induced extracellular pressure (CEP) component of IMP as prescribed in some studies (Algranati et al., 2010; Mynard and Smolich, 2016). As such, the IMP waveform is dependent on the active constitutive model in Eqs. 18–20, where active fiber stress is prescribed to peak at *t*_0_ = 275ms (corresponding to late systole). We note that fiber stress predicted by other active constitutive models (e.g., Figure 8 of Bovendeerd et al., 1992) peak at early to mid-systole. In our model, the resultant IMP waveform at the endocardium (Figure 3C) peaks at end-systole (Figure 3C), which is consistent with experimental measurements at a fractional wall thickness of 0.7 (Figure 5B of Heineman and Grayson, 1985). These features led to the sub-endocardial coronary flow rate reaching its lowest value at late-systole (Figure 3D), although we note that the timepoint of the lowest flow varies in measurements (Mizutani et al., 1993; Ofili et al., 1993; Hiramatsu et al., 1994; Hadjiloizou et al., 2008; Fan et al., 2021). In fact, Westerhof (1990) had also rationalized that *“intramyocardial pressure is not always related to (left) ventricular pressure, as is generally assumed.”* For that reason, the IMP/LVP ratio at the endocardium may not be equal to 1 and varies between 0.7 and 1.3 for all the cases in this study (Figures 5–9). This may also explain the wide variation of the IMP/LVP ratio measured in the experiments that are often conducted under different conditions, with some measuring a ratio less than 1 (e.g., ratio is 0.85 in Figure 5 in Brandi and Mcgregor, 1969) and others measuring a ratio exceeding 1 (Rabbany et al., 1989). The indications from experimental measurements that the peak IMP/LVP ratio is increased with contractility as mentioned by Westerhof (1990) is also consistent with our findings, where the ratio increases from 0.8 to 1.1 with an increase in contractility (Figure 5). We note, however, that the variation of IMP/LVP ratio seen in the experiments may also be due to differences in techniques (Westerhof, 1990).

Not only is the transmural variation of *p* in a pressurized LV similar to experimental measurements (Figure 4A), prescribing *p* as IMP also enables the model to predict **(1)** similar effects of contractility impeding coronary flow under isobaric conditions (zero-cavity pressure) in a beating LV as found in Krams et al. (1989a), **(2)** the persistence of systolic IMP in an empty heart (Baird et al., 1972), where a high peak IMP (70–124 mmHg) in the unpressurized heart is found (Salisbury et al., 1962), and **(3)** reduced coronary flow at the endocardium compared to the epicardium in a contracting low-pressurized heart (see Table 2 of Doucette et al., 1993; see Supplementary Appendix D). The latter two features cannot be produced by prescribing CEP as the IMP. These two features can only be produced with shortening-induced intracellular pressure (SIP) and varying elastance (VE) components of the IMP as described in some studies (Algranati et al., 2010; Mynard and Smolich, 2016). As such, *p* embodies features of the CEP, VE, and SIP components of IMP. Also, for this reason, IMP is not directly related to LV pressure as shown in Figures 5–8.

### Transmural Distribution of Coronary Perfusion

By prescribing the hydrostatic pressure as the extravascular force acting on the coronary vessels, the model is able to predict the transmural distribution of passive coronary flow under normal and isobaric conditions (Supplementary Appendix D) that are in agreement with the experiments (Krams et al., 1989b). Specifically, our model predictions of the coronary flow rate waveforms at different transmural locations (Figure 3D) are comparable with that of a previous modeling study (Mynard et al., 2014). In that study, it was found that the flow rate at the endocardial (*R* = 0) and the mid-endo (*R* = 1/3) regions are largely negative during systole because the IMP is higher in the endocardial region than that in the epicardial region (*R* = 1) in systole. We also calculated the coronary flow velocity at the endocardium with different relaxation time constant τ and compared it with experimental measurements (Ofili et al., 1993; Toyota et al., 2005; Supplementary Figures F1D–F). It must be noted that the measurements are associated with flow measured in the sub-endocardial micro-vessels under regulated conditions (Toyota et al., 2005) and in the large coronary arteries under fully vasodilated condition (Ofili et al., 1993), which are not the same as that in the model. Nevertheless, notwithstanding the difference in magnitude that could arise because of the measurement conditions, vessel size etc., the comparison show that the model predicted coronary flow velocity waveform shares some features with the experimental measurements with flow velocity reaching a negative peak value in systole and a positive peak value during the isovolumic relaxation phase that is sharper in the micro-vessels. There are, however, some notable key difference between the measurement and model prediction is a higher and sharper positive peak flow velocity that occurs earlier (*t* ∼ 300 ms) in the isovolumic relaxation phase than that in the experiments (*t* ∼ 550 ms). This difference is likely due to differences in τand the fact that we did not consider the vessels upstream of the microvascular network. Increasing τ reduces the peak flow velocity with the changes largely occurring at the beginning of diastole. With the addition of a vessel (represented by a capacitor and resistance) upstream on the other hand (Supplementary Figure G1A), the peak flow velocity shifted right in the waveform at the endocardium and occurs later during isovolumic relaxation (instead at the onset) (Supplementary Figure G1B). Similarly, peak flow rate at the mid-epicardium and epicardium also shifted to the right in the waveform. This shift resulted in a higher flow rate during diastole than systole (Supplementary Figure G2B) as opposed to when vessels upstream are not considered (Supplementary Figure G2A). Differences in the coronary flow waveform may also be attributed to some disparity in the IMP waveform.

As a result of the transmural variation in IMP, the model predicts that the total passive coronary flow is lower in the endocardial than the epicardial regions. The predicted ratio of coronary flow in the endocardial region to that in the epicardial region *Q*_*endo*_/*Q*_*epi*_ under passive condition is 0.59 in the baseline case (Figure 4B). This result is comparable to the range of 0.64–0.90 measured in a normal canine model (Bin et al., 2003) under vasodilated conditions. We note, however, that in the presence of coronary regulation, *Q*_*endo*_/*Q*_*epi*_ is greater than 1.0 as measured in some studies (Bache et al., 1981a). With increasing vasodilation induced pharmacologically (Domenech and MacLellan, 1980) or by exercise (Duncker et al., 1998), *Q*_*endo*_/*Q*_*epi*_ was found to be reduced, however, and have values below unity that is in agreement with our findings.

The model predicts that coronary flow is increased across all transmural locations with an increase in contractility *T*_*max*_, afterload and preload. The increase in coronary flow is less at the endocardium than at the epicardium, especially with an increase in contractility (Figure 5D). This result can be explained by the presence of a competition between the increased perfusion pressure (that promote flow) and increased IMP (that impedes flow) at the endocardium. At the epicardium, coronary flow is controlled mainly by the perfusion pressure as IMP is small. Increasing contractility *T*_*max*_ therefore results in a more severe redistribution of coronary flow in the transmural layers of the LV wall. The transmural gradient of coronary flow is increased with increasing *T*_*max*_. This is in contrast to the increase in preload and afterload, where the gradient is approximately the same. Unlike the effects of contractility and loading conditions, increasing LV wall thickness and cavity volume (with constant afterload and preload) reduces coronary perfusion across the myocardial wall (Figures 8D, 9D). In addition, *Q*_*endo*_/*Q*_*epi*_ is also reduced from 0.80 to 0.45 with increasing wall thickness (from 0.53 to 1.73 cm), and 1.1 to 0.44 with dilating LV cavity volume (from EDV of 108 to 114 ml). These results are broadly in agreement with experiments, where a marked decrease in *Q*_*endo*_/*Q*_*epi*_ ratio is associated with a thickening of the myocardial wall induced by severe stenosis (Bin et al., 2002, 2003) and LV hypertrophy (Bache et al., 1981b) (*Q*_*endo*_/*Q*_*epi*_ is reduced from 0.93 to 0.86). Our findings therefore suggest that the reduction in *Q*_*endo*_/*Q*_*epi*_ associated with adverse remodeling seen in the experiments may be explained in part by a change in the geometry in addition to any other changes that may have occurred with the remodeling of coronary vasculature.

### Transmural Distribution of Myocardial Work Density

It is well-established that global myocardial work density can be indexed by the pressure-volume area, which is linearly correlated to the total myocardial oxygen consumption MVO_2_ (Suga, 1979). At the local level, however, that relationship is not clear though it has been shown to be correlated to cardiac metabolism in a recent study (Duchenne et al., 2019). By indexing local myocardial work density *W_f_* with the area in the average fiber stress-strain *S*_*ff*_−*E*_*ff*_loop, we show that the model predicts a transmural distribution of *W_f_* across the LV wall that is relatively constant from the endocardium (*R* = 0) to the mid-wall, but reduces linearly from the mid-wall to epicardium (*R* = 1) (Figure 4C). Our model predicts that *W_f_* is about 87% higher at the endocardium than epicardium. Because peak myofiber strain is fairly uniform, ranging from 0.1 to 0.12 transmurally that is consistent with experimental measurements (van der Vusse et al., 1990; Omens et al., 1991, 1993), the variation in *W_f_* is largely attributed to a smaller peak myofiber stress at the epicardium compared to that at the endocardium (Figure 4D). While previous studies have shown that peak myofiber stress is largely uniform across the ventricular wall (van der Vusse et al., 1990; Carruth et al., 2016), the uniformity is concentrated largely between the base and mid-ventricle (Guccione et al., 1995) or applies only to a cylindrical idealization of the LV (Arts et al., 1979; Chadwick, 1982). At the apex, however, peak myofiber stress is larger at the endocardial than the epicardial region (Bovendeerd et al., 1994; Guccione et al., 1995) even though there is more uncertainty associated with this result because of the lack of physiological data (e.g., myofiber distribution) at that region (Bovendeerd et al., 1992). As such, when averaging the transmural variation of *W*_*f*_over the whole LV, our results showing that *W_f_* is higher at the endocardium than the epicardium is largely consistent with previous models.

Due to technical difficulties, there are no direct experimental measurements of the transmural myocardial work that are available for comparison with our model prediction (see van der Vusse et al., 1990 for a review). Although it has been postulated earlier that little transmural differences in myocardial work (in the order of 10–20%) exist, experimental measurements of quantities related to myocardial work have not provided any definite conclusion regarding the existence or absence of a transmural gradient in *W_f_* (van der Vusse et al., 1990). Nevertheless, the transmural variation of *W_f_* that we found here is consistent with some experiment findings of the transmural pattern of several quantities that are reflective of myocardial work density. First, this transmural variation of *W*_*f*_ is consistent with experiments showing that subendocardial oxygen consumption per unit weight is about 20% higher than that in the epicardium (Weiss, 1979), with a steeper change occurring between epicardium to the mid-wall than that between the mid-wall and endocardium. Second, the transmural variation of *W_f_* is also similar to that of coronary blood flow under normal regulated conditions, where *Q*_*endo*_ is about 10% (Bache et al., 1981a) to 50% higher than *Q_epi_* (Duncker et al., 1998). This result suggests that coronary flow is regulated in response to the work density distribution. Third, and interestingly, the transmural variation of *W*_*f*_ is also in agreement with the findings that the myocyte volume at the endocardium is about 20% higher than that at the epicardium in normal human heart (Vliegen et al., 1987). The larger myocytes found at the endocardium is reflective of a higher workload in the endocardial region (Waring et al., 2014). Although differing in magnitude, the findings that the transmural variation of *W_f_* is consistent with that of several cardiac workload indices, therefore, support the use of fiber stress-strain area predicted by the model as an index of myocardial work density.

With the exception of wall thickness, the model predicts that *W_f_* is increased monotonically across all transmural locations with an increase in contractility *T*_*max*_, afterload, preload and LV cavity volume. These results are broadly consistent with previous studies. Specifically, the effects of preload and afterload are consistent with a modeling study (Beyar and Sideman, 1986) showing that myocardial work density is increased with an increased gradient between the endocardial and epicardial regions when preload and afterload are elevated (Figures 6E, 7E). Moreover, the transmural gradient of adenosine triphosphate (ATP) production, a measure of myocardial work density (Aasum, 2011), was found to be increased by about 5% with increased contractility in a canine study (Bache et al., 1994), a trend that is consistent with our model prediction that the transmural gradient of *W_f_* (ratio of endocardial to epicardial *W_f_*) is increased by about 2.8% with a 215% increase in contractility (from baseline) (Figure 5E). Furthermore, it was also found in the same study that ATP was reduced at both endocardium and epicardium in concentric hypertrophy (Bache et al., 1994). This is consistent with our model prediction that the myocardial work density is reduced when wall thickness is increased by 53.1% from the baseline case. That study also found that the ratio of ATP production between endocardium to epicardium ratio was slightly increased by about 3.5%, which is similar to the endo:epi ratio of *W_f_* predicted by the model (Figure 8E). Taken together, these findings suggest that the increase in wall thickness has a compensatory effect of reducing *W_f_* in hypertrophy.

### Transmural Distribution of Work Density-Perfusion Mismatch

The ratio of myocardial work density *W_f_* to coronary blood flow *Q*, which we refer to as the work density-perfusion mismatch index (*W*_*f*_/*Q*), provides a measure of the relative change of myocardial demand with respect to myocardial supply under maximal hyperemia (i.e., maximal vasodilation). While *W*_*f*_/*Q* is somewhat similar to the ratio between diastolic pressure time index (DPTI) (myocardial supply) and tension time index (TTI) (myocardial demand) that is typically determined experimentally to relate the relative global changes of myocardial demand and supply (Buckberg et al., 1972; Hoffman and Buckberg, 1978) or the subendocardial viability ratio (Tsiachris et al., 2012), the fact that myocardial demand is a “virtual parameter” that cannot be measured experimentally (Heusch, 2019) calls into question if the DPTI/TTI ratio is truly indicative of myocardial demand/supply mismatch. This is because contractile function is always tied to myocardial blood flow experimentally (i.e., perfusion-contraction matching) (Ross, 1991). As mentioned by Heusch (2019), *“Regional myocardial blood flow and contractile function in ischemic myocardium are well matched, and there is no evidence for an oxygen supply/demand imbalance.”* Unlike in experiments, however, one can decouple contractile function from myocardial blood flow *in silico* (as we have done here) to evaluate if the ratio of myocardial work density (that is unconstrained by myocardial blood flow) to perfusion (that is still affected by LV mechanics) is worsened or improved under different loading and/or geometrical conditions. An increase in *W*_*f*_/*Q* would imply that the myocardium is more susceptible to ischemia and vice-versa.

Here, we show that because myocardial demand (Figures 5E, 7E) is increased more than the increase in coronary flow (Figures 5D, 7D) at the endocardium (*R* = 0) than epicardium (*R* = 1) when contractility, preload, wall thickness and LV cavity volume are increased, myocardial demand-supply imbalance is exacerbated at the endocardium (Figures 5F, 7F). Further, the gradient of the linear variation of mismatch increases with increasing contractility, with the endo:epi ratio of the mismatch increasing from 1.37 to 2.81. This result is in agreement with the clinical observation that endocardium is more vulnerable to ischemia than epicardium (Algranati et al., 2011) as well as in animal studies showing that increasing contractility (by injecting isoproterenol, an inotropic agent) can cause endocardial ischemia (Buckberg and Ross, 1973). Similarly, this result suggests that the increase in preload and wall thickness associated with heart failure with preserved ejection fraction (HFpEF), may also produce myocardial demand-supply imbalance, particularly at the endocardium. This result is consistent with a canine study (Archie, 1975) showing that ischemia is induced at the endocardium with an increase in preload and a reduction in coronary flow reserve at the endocardium in concentric LV hypertrophy (Vatner and Hittinger, 1993). This finding also suggests that besides coronary endothelial dysfunction, an increase in preload and wall thickness may play a role in the reduction of coronary flow reserve in HfpEF (Mohammed et al., 2016). On the other hand, the worsening of work density-perfusion index with an increase in cavity volume is also consistent with the reduction of coronary flow reserve found in patients with dilated cardiomyopathy but with normal coronary arteries (Inoue et al., 1993). Taken together, these results suggest that changes in ventricular loading conditions, myocardial contractility, as well as pathological changes in geometry all contribute to the worsening of myocardial work density-perfusion mismatch and may play a role in the overall reduction in coronary flow reserve in heart failure.

Since all heart failure, by definition, involves some degree of myocardial oxygen supply/demand mismatch, the work density-perfusion mismatch ratio *W*_*f*_/*Q* can have significant implication in our understanding, management, and treatment of heart diseases. Specifically, regional *W*_*f*_/*Q* can be computed to assess key contributors of mismatch in heart diseases and suggest how this ratio can be improved to reduce subendocardial hypoperfusion or ischemia. For example, in HFpEF where both preload and afterload are typically increased (especially those involving hypertension (Tam et al., 2017)), the study suggests that treatment reducing preload (e.g., by diuresis) can better improve the work density-perfusion mismatch than those altering afterload. Moreover, future work that quantify a critical *W*_*f*_/*Q* for the onset of ischemia (e.g., as indicated by the lactate level) may help in assessing disease progression and optimizing treatment, such as managing the supposedly increase in RV work density by LV assist device in patients (Guglin et al., 2020).

### Limitations

There are some limitations associated with this study. First, the current model is limited to an ellipsoidal LV geometry that can be extended to subject-specific geometries to increase its realism. Second, the same coronary network was implanted at different transmural locations across the LV wall, which does not consider differences in morphological characteristics (i.e., diameters, lengths, branching pattern, etc.) of transmural beds or any potential collateral blood flow across the wall. This limitation can be overcome in future studies by incorporating a full-scale coronary network with differences in transmural morphology and possible collaterals. Third, regional differences in diastolic and systolic time fractions between endocardium and epicardium are not considered in this study. In principle, the myocardium contracts first in the endocardium, which may give rise to a longer systolic time fraction in the endocardium than the epicardium (Goodwill et al., 2018). This limitation can be overcome by taking into account cardiac electrophysiology in the model. Fourth, the model treats the myocardial tissue as an incompressible material, and does not take into account changes in LV wall volume (up to 15%) (May-Newman et al., 1994) due to blood flowing into the myocardium. To address this limitation, the model can be improved by describing the myocardial tissue as a poroelastic material or compressible material. Fifth, the boundary condition in which the base is constrained from moving out of plane is more similar to a Langendorff preparation than it is to a heart *in vivo*, where the base is moving out of plane and the apex has little motion. These boundary conditions have been applied in previous models (Guccione et al., 1995; Balaban et al., 2018; Zhang et al., 2018, 2021; Shavik et al., 2021). As mentioned in Guccione et al. (2003), however, stress and strain would still be largely similar because these quantities are independent of rigid body motion. Moreover, we have also shown that the model is able to simultaneously predict LV pressure, volume and strain waveforms that are close to those measured *in vivo*. More realistic boundary conditions (Strocchi et al., 2020b) can be imposed in future. Sixth, we have not investigated the interactions and contributions of different loading conditions (preload and afterload), LV wall thickness and cavity volume on transmural myocardial work and perfusion. Only the isolated effects of these factors are considered. A global sensitivity analysis (Strocchi et al., 2020a; Rodero et al., 2021) can be performed in future studies to evaluate the interactions and contributions of these factors to patient-specific changes in coronary hemodynamics and LV mechanics under different pathological conditions. Seventh, we did not consider vessels upstream of the microvascular network, which in a preliminary study (Supplementary Figure G1), shows that it affects the location of the positive peak in the coronary flow velocity waveform. Eighth, IMP implemented as a Lagrange multiplier may not be able to produce all features found in measurements, which can be improved by considering more mechanisms. Last, coronary flow regulation is not taken into account here and so, the model is only able to predict the transmural distribution of coronary flow under fully dilated conditions. Future studies will consider regulation mechanisms to directly predict coronary flow reserve.

## Summary and Conclusion

We developed a computational framework that couples an LV FE model and coronary perfusion in a closed-loop manner. Model predictions of the transmural distribution of myocardial work density, coronary flow under vasodilated condition, IMP as well as global hemodynamics and strain waveforms all show a good agreement with experimental measurements. The model predicted coronary flow (under passive condition) is increased with increasing LV contractility, afterload and preload at all transmural positions, but is reduced with increasing wall thickness and cavity volume. The model also predicted myocardial work density is increased with increasing LV contractility, afterload, preload and cavity volume at all transmural positions, but is reduced with increasing wall thickness. Taken together, the model predicted that the ratio of myocardial work density to coronary flow (work density-perfusion mismatch) is increased with increasing LV contractility, preload, wall thickness and cavity volume. The increase is larger in the endocardium compared to the epicardium, suggesting an increase in subendocardial vulnerability to ischemia with these factors.

## Data Availability Statement

The data and code that support the findings of this study are available at https://bitbucket.org/FanLei1/transmural_distribution_wq/src/master/.

## Author Contributions

All authors contributed to the conception and design of the study, contributed to the analysis of results, drafted the manuscript, revised it critically for important intellectual content, approved final version of the manuscript, and significantly contributed to the submitted work.

## Conflict of Interest

The authors declare that the research was conducted in the absence of any commercial or financial relationships that could be construed as a potential conflict of interest.

## Publisher’s Note

All claims expressed in this article are solely those of the authors and do not necessarily represent those of their affiliated organizations, or those of the publisher, the editors and the reviewers. Any product that may be evaluated in this article, or claim that may be made by its manufacturer, is not guaranteed or endorsed by the publisher.

## Footnotes

1
http://www.llnl.gov/casc/sundials/
