# Supplementary material for: Transmural Distribution of Coronary Perfusion and Myocardial Work Density Due to Alterations in Ventricular Loading, Geometry and Contractility
Source: Front Physiol. 2021 Nov 24;12:744855. doi: 10.3389/fphys.2021.744855 (PMC8652301; doi:10.3389/fphys.2021.744855)
Supplement: Supplementary file 1 [file Data_Sheet_1.docx]

**Fig A1**: Effects of mesh size on **a.** Pressure waveforms; **b.** LV pressure-volume loop; **c.** Transmural distribution of IMP; **d.** $E_{cc}$; and **e.** $E_{ll}$. “nele” denotes to the number of elements.

**Fig D1**: Simulation results of an isolated beating LV under isobaric zero LV cavity pressure condition. **A.** Inlet pressure waveform; **b.** Coronary flow over a cardiac cycle in the endocardium (*R*=0) with different contractility; **c.** Transmural distribution of total coronary flow over a cardiac cycle; **d.** Transmural distribution of IMP at maximum contraction.

**Fig E1**: Fiber stress strain relationship at different transmural locations with different **a.** contractility; **b.** afterload; **c.** preload; **d.** wall thickness; **e.** cavity volume.

**Fig F1**: Effects of isovolumic relaxation rate $\tau$ on **a.** Pressure waveforms with measured LV (Shavik et al., 2019) and arterial pressure waveforms (Ofili et al., 1993); **b.** LV volume waveforms with measurements (Shavik et al., 2019); **c.** coronary flow rate waveforms at the endocardium; **d.** coronary flow velocity waveforms at the endocardium with measurement (Toyota et al., 2005); **e.** coronary flow velocity waveforms at the endocardium with measurement (Ofili et al., 1993); **f.** normalized coronary flow velocity at baseline case with measurements. **G.** preliminary results of coronary flow velocity at endocardium with one more set of compliance and resistor**; h.** transmural distribution of total coronary flow. “base”, “+$\tau$” and “++$\tau$” denote $\tau=25$ms, 45ms and 105ms.

**Fig G1:** **a**. Schematic of the 400-vessel network with the addition of a vessel (represented by a capacitor and resistance) upstream; **b.** The effects of the addition of a vessel (denoted by “1ele”) in the upstream on coronary flow velocity at the endocardium.

**Fig G2:** Coronary flow rate waveforms at the mid-epicardium and epicardium in the **a.** baseline case and **b**. case with an additional upstream vessel.

**Appendix A** Convergence study

Increasing number of elements (“*nele”*) from 1159 to 54773 by reducing the mesh size shows that pressures, LV pressure-volume loop, transmural distribution of IMP, and myocardial strain converged when the number of elements is greater and equal to 4353 (**Fig A1)**.

**Appendix B** Parameters used for a model of systemic circulation

The parameters used in the model are listed in **Table B1-B3**.

**Table B1**: Parameters used in systemic circulation.

| Parameter | Unit |  |
| --- | --- | --- |
| $R_{ao}$ | mmHg ms/ml | $5.00\times{10}^{2}$ |
| $R_{ven}$ | mmHg ms/ml | $1.00\times{10}^{2}$ |
| $R_{mv}$ | mmHg ms/ml | $2.00\times{10}^{2}$ |
| $R_{a,p}$ | mmHg ms/ml | $1.80\times{10}^{4}$ |
| $R_{a,d}$ | mmHg ms/ml | $1.06\times{10}^{5}$ |
| $C_{ven}$ | ml/mmHg | $2.80\times{10}^{-1}$ |
| $C_{a,p}$ | ml/mmHg | $3.20\times{10}^{-3}$ |
| $C_{a,d}$ | ml/mmHg | $3.30\times{10}^{-2}$ |
| $V_{ven0}$ | ml | $3.37\times{10}^{3}$ |
| $V_{LA0}$ | ml | $1.00\times10$ |
| $V_{a,p0}$ | ml | $3.60\times{10}^{2}$ |
| $V_{a,d0}$ | ml | $4.00\times10$ |
| $V_{ven}$ | ml | $3.70\times{10}^{3}$ |
| $V_{LA}$ | ml | $1.20\times10$ |
| $V_{a,p}$ | ml | $7.40\times{10}^{2}$ |
| $V_{a,d}$ | ml | $1.00\times{10}^{2}$ |

**Table B2**: Parameters used in time-varying elastance model of left atrium.

| Parameter | Unit |  |
| --- | --- | --- |
| $T_{max,LA}$ | ms | $1.90\times{10}^{-2}$ |
| $A_{LA}$ | mmHg | $2.67$ |
| $B_{LA}$ | ml^-1^ | $3.77\times{10}^{-1}$ |
| $\tau_{LA}$ | ms | $25$ |
| $E_{es,LA}$ | mmHg/ml | $1.00\times10$ |

Parameters across all orders in the coronary circulation are obtained by a quadratic fit of the parameter $A_{p}$, and linear fits to $\varphi_{p}$ and $C_{p}$ through all data points.

**Table B3**: Parameters used in a finite element model of the left ventricular.

| Parameter | Unit |  |
| --- | --- | --- |
| $T_{max}$ | kPa | $1.30\times{10}^{5}$ |
| ${Ca}_{0}$ | $\mu$M | $4.35$ |
| ${Ca}_{50}$ | $\mu$M | $1.00\times{10}^{2}$ |
| ${{(Ca}_{0})}_{max}$ | $\mu$M | $4.35$ |
| $T_{trans}$ | ms | $3.00\times{10}^{2}$ |
| $t_{0}$ | ms | $275$ |
| $l_{0}$ | $\mu$m | $1.58$ |
| $l$ | $\mu$m | 1.85 |
| $b$ | ms | 1675 |
| $B$ | $\mu$m^-1^ | $4.75$ |
| $b_{ff}$ | - | $2.90\times10$ |
| $b_{fx}$ | - | $1.33\times10$ |
| $b_{xx}$ | - | $2.66\times10$ |

**Table B4**: Parameters used in coronary circulation.

| Order | $A_{p}$($\mu$m) | $B_{p}$($\mu$m) | $\varphi_{p}$(mmHg)$Type equation here.$m) | $C_{p}$(mmHg) |
| --- | --- | --- | --- | --- |
| 0 | 3.20 | 2.43 | 0 | 19.28 |
| 1 | 4.51 | 3.59 | 0 | 17.24 |
| 5 | 35.02 | 10.9 | 0.61 | 20.11 |
| 6 | 51.38 | 16.41 | 1.88 | 14.23 |
| 6 | 85.53 | 32.17 | 1.64 | 21.24 |
| 7 | 133.52 | 51.6 | 0.96 | 23.54 |
| 10 | 829.6 | 401.52 | 30.39 | 3.35 |

**Appendix C Finite element formulation of the left ventricle**

Finite element formulation of the LV can be generalized from the minimization of the following Lagrangian function

$\mathcal{L}\left( \boldsymbol{u,}p,P_{\mathrm{cav}},\boldsymbol{c}_{1},\boldsymbol{c}_{2} \right)=\int_{\Omega_{0}} W\left( \boldsymbol{u} \right)dV-\int_{\Omega_{0}} p\left( J-1 \right)dV-P_{\mathrm{cav}}\left( V_{\mathrm{cav}}\left( \boldsymbol{u} \right)-V \right)-\boldsymbol{c}_{1}\boldsymbol{\cdot}\int_{\Omega_{0}} \boldsymbol{u} dV-\boldsymbol{c}_{2}\boldsymbol{\cdot}\int_{\Omega_{0}} \boldsymbol{X}\times\boldsymbol{u} dV.$ (C1)

In the above equation, $\boldsymbol{u}$ is the displacement field,$P_{\mathrm{cav}}$ is the Lagrange multiplier to constraint the cavity volume $V_{\mathrm{cav}}(\boldsymbol{u})$to a prescribed value $V$ (Pezzuto and Ambrosi, 2014), $p$ is a Lagrange multiplier to enforce incompressibility of the tissue (i.e., Jacobian of the deformation gradient tensor$J=1$), and both $\boldsymbol{c}_{1}$ and $\boldsymbol{c}_{2}$ are Lagrange multipliers to constrain rigid body translation (i.e., zero mean translation) and rotation (i.e., zero mean rotation) (Pezzuto et al., 2014). The LV cavity volume $V_{\mathrm{cav}}$ is a function of the displacement $\boldsymbol{u}$ and is defined by

$V_{\mathrm{cav}}\left( \boldsymbol{u} \right)= \int_{\Omega_{inner}} dv=-\frac{1}{3}\int_{\Gamma_{inner}} \boldsymbol{x}\boldsymbol{.} \boldsymbol{n} da ,$ (C2)

where $\Omega_{inner}$ is the volume enclosed by the inner surface $\Gamma_{inner}$ and the basal surface at *z* = 0, and $\boldsymbol{n}$ is the outward unit normal vector.

Pressure-volume relationship of the LV required in the lumped parameter circulatory model was defined by the solution obtained from minimization of the function. Taking the first variation of the function in Eq. (C2) leads to the following expression:

$\delta\mathcal{L}\left( \boldsymbol{u},p,P_{\mathrm{cav}},\boldsymbol{c}_{1},\boldsymbol{c}_{2} \right)=\int_{\Omega_{0}} \boldsymbol{(P-}p\boldsymbol{F}^{\boldsymbol{-T}}):\nabla\delta\boldsymbol{u} dV-\int_{\Omega_{0}} \delta p\left( J-1 \right)dV-P_{\mathrm{cav}}\int_{\Omega_{0}} cof\left( \boldsymbol{F} \right):\nabla\delta\boldsymbol{u} dV-\delta P_{\mathrm{cav}}\left( V_{\mathrm{cav}}\left( \boldsymbol{u} \right)-V \right)-{\delta\boldsymbol{c}}_{1}\boldsymbol{\cdot}\int_{\Omega_{0}} \boldsymbol{u} dV-\delta\boldsymbol{c}_{2}\boldsymbol{\cdot}\int_{\Omega_{0}} \boldsymbol{X}\times\boldsymbol{u} dV-\boldsymbol{c}_{1}\boldsymbol{\cdot}\int_{\Omega_{0}} \boldsymbol{u} dV-\boldsymbol{c}_{2}\boldsymbol{\cdot}\int_{\Omega_{0}} \boldsymbol{X}\times\delta\boldsymbol{u} dV.$ (C3)

In Eq. (C3), $\boldsymbol{P}$ is the first Piola Kirckhoff stress tensor, $\boldsymbol{F}$ is the deformation gradient tensor,$\delta\boldsymbol{u}$,$(\delta p$, ${\delta P}_{\mathrm{cav}})$ ${\delta\boldsymbol{c}}_{1}$ $\delta\boldsymbol{c}_{2}$are the variation of the displacement field, Lagrange multipliers for enforcing incompressibility and volume constraint, zero mean translation and rotation, respectively. The Euler-Lagrange problem then becomes finding $\boldsymbol{u}{\boldsymbol{\in}H}^{1}\left( \Omega_{0} \right), p\boldsymbol{\in}L^{2}\left( \Omega_{0} \right),P_{\mathrm{cav}}\mathbb{\in R},\boldsymbol{c}_{1}\boldsymbol{\in}\mathbb{R}^{\boldsymbol{3}},\boldsymbol{c}_{2}\boldsymbol{\in}\mathbb{R}^{\boldsymbol{3}}$that satisfies

$\delta\mathcal{L}\left( \boldsymbol{u},p,P_{\mathrm{cav}},\boldsymbol{c}_{1},\boldsymbol{c}_{2} \right)=0$ (C4)

and $\left. \boldsymbol{u}\left( x,y,0 \right)\boldsymbol{.n} \right|_{base}\boldsymbol{=}0$ (for constraining the basal deformation to be in-plane) $\forall\delta\boldsymbol{u}{\boldsymbol{\in}H}^{1}\left( \Omega_{0} \right), \delta p\boldsymbol{\in}L^{2}\left( \Omega_{0} \right),{\delta P}_{\mathrm{cav}}\mathbb{\in R},{\delta\boldsymbol{c}}_{1}\boldsymbol{\in}\mathbb{R}^{\boldsymbol{3}},\delta\boldsymbol{c}_{2}\boldsymbol{\in}\mathbb{R}^{\boldsymbol{3}}\boldsymbol{.}$ In the implementation, the displacement field, $\boldsymbol{u}$, is discretized by quadratic elements and the Lagrange multiplier, $p$, is discretized by linear elements.

**Geometry of the LV**

The LV geometry was idealized using a half prolate ellipsoid that was discretized with 4353 tetrahedral elements (see Appendix A1 for mesh-convergence study). The helix angle associated with the myofiber direction $\mathbf{e}_{f_{0}}$was varied with a linear transmural variation from $60^{\circ}$ at the endocardium to $-60^{\circ}$ at the epicardium in the LV wall based on previous experimental measurements (Streeter et al., 1969).

**Constitutive law of the LV**

An active stress formulation was used to describe the LV’s mechanical behavior in the cardiac cycle. In this formulation, the stress tensor $\boldsymbol{P}_{\mathrm{LV}}$ can be decomposed additively into a passive component $\boldsymbol{P}_{LV, p}$ and an active component $\boldsymbol{P}_{LV, a}$ (i.e., $\boldsymbol{P}_{\mathrm{LV}}\boldsymbol{=}\boldsymbol{P}_{LV, a}\boldsymbol{+}\boldsymbol{P}_{LV, p}\boldsymbol{)}$. The passive stress tensor was defined by $\boldsymbol{P}_{LV, p}\boldsymbol{=}{dW}_{\mathrm{LV}}/d\boldsymbol{F}_{\mathrm{LV}}$, where $\boldsymbol{F}_{\mathrm{LV}}$ **i**s the deformation gradient tensor and $W_{\mathrm{LV}}$ is a strain energy function of a Fung-type transversely-isotropic hyperelastic material (Guccione et al., 1991) given by

$W_{\mathrm{LV}}= \frac{1}{2}C\left( e^{Q}-1 \right),$ (C5a)

where,

$Q=b_{ff}E_{ff}^{2}+b_{xx}\left( E_{ss}^{2}+E_{nn}^{2}+E_{sn}^{2}+E_{ns}^{2} \right)+b_{fx}\left( E_{fn}^{2}+E_{nf}^{2}+E_{fs}^{2}+E_{sf}^{2} \right).$ (C5b)

In Eq. (C5), *E_ij_* with (*i, j*) ∈ (*f, s, n*) are components of the Green-Lagrange strain tensor$\boldsymbol{E}_{\mathrm{LV}}$ with *f*, *s*, *n* denoting the myocardial fiber, sheet, and sheet normal directions, respectively. Material parameters of the passive constitutive model are denoted by *C*, *b_ff_*, *b_xx_* and *b_fx_*. The active stress $\boldsymbol{P}_{LV, a}\boldsymbol{=}\boldsymbol{F}_{\mathrm{LV}} \boldsymbol{S}_{LV, a}$ was calculated along the local fiber direction using the active constitutive relationship given in Eq. (18) – (20).

**Appendix D** Effects of coronary flow under isobaric condition

Simulations were performed on an isolated beating LV with the coronary microvascular networks at 3 different levels of myocardial contractility (i.e., $T_{max}=65, 130, 205\mathrm{kPa}$). Traction free boundary conditions (i.e., zero cavity pressure) were imposed on the LV while the microvascular networks were prescribed with a periodic inlet pressure boundary condition (**Fig D1a**) and a constant outlet pressure of 20 mmHg. These boundary conditions mimic the experimental conditions of an isobaric, low loaded beating LV, and a time-periodic perfusion pressure. Under these conditions, the computational framework predicted the coronary flow rate was reduced during systole, with the greatest reduction found in the endocardial layer (*R*=0). The magnitude of this reduction increased with myocardial contractility (**Fig D1b**). Correspondingly, total coronary flow over a cardiac cycle varied and increased transmurally from the endocardium (*R*=0) to the epicardium (*R*=1). At each myocardial layer, the total flow was decreased with increasing myocardial contractility, where endocardium (*R*=0) has the greatest decrease (**Fig D1c**). We note that the IMP (i.e., Lagrange multiplier) also decreased transmurally with a linear variation from the endocardium (*R*=0) to the epicardium (*R*=1) even though the LV cavity pressure is zero (**Fig D1d**).

**Appendix E** Myocardial work in transmural location

The $S_{ff}-E_{ff}$ loops obtained in sensitivity analyses by varying contractility, afterload, preload, wall thickness, and cavity volume are presented in **Fig E1**.

**Appendix F** Effects of isovolumic relaxation time constant on the pressure and coronary flow velocity waveforms.

The effects of isovolumic relaxation time constant$\tau$ on pressure, LV volume and coronary flow rate and velocity waveforms are shown in **Fig F1** with cases “base”, $"+\tau"$, $"++\tau"$ corresponding to simulation ran with$\tau=25ms,$ $\tau=45ms$ and $\tau=105ms,$ respectively. A comparison with experimental measurements of the coronary flow rate waveform was also made in those figures

**Appendix G** Preliminary studies on the inclusion of a vessel upstream of the microvascular network

The schematic of the 400-vessel network with the addition of a vessel (represented by a capacitor and resistor) upstream is shown in **Fig. G1a.** The effects of an upstream vessel on coronary flow velocity at the sub-endocardium is shown in **Fig. G1b**. Coronary flow rate waveform at the mid- and sub-epicardium with and without an upstream vessel is shown in **Fig. G2b** and **Fig. G2a**, respectively.

**Fig A1**

**
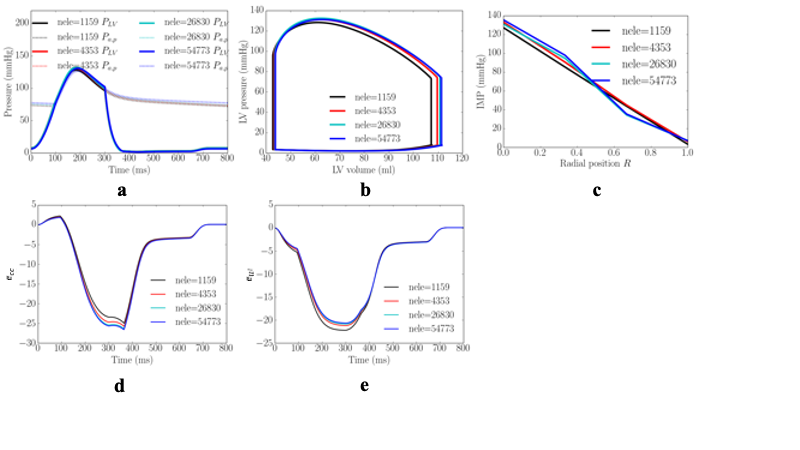
**

**Fig D1**

**
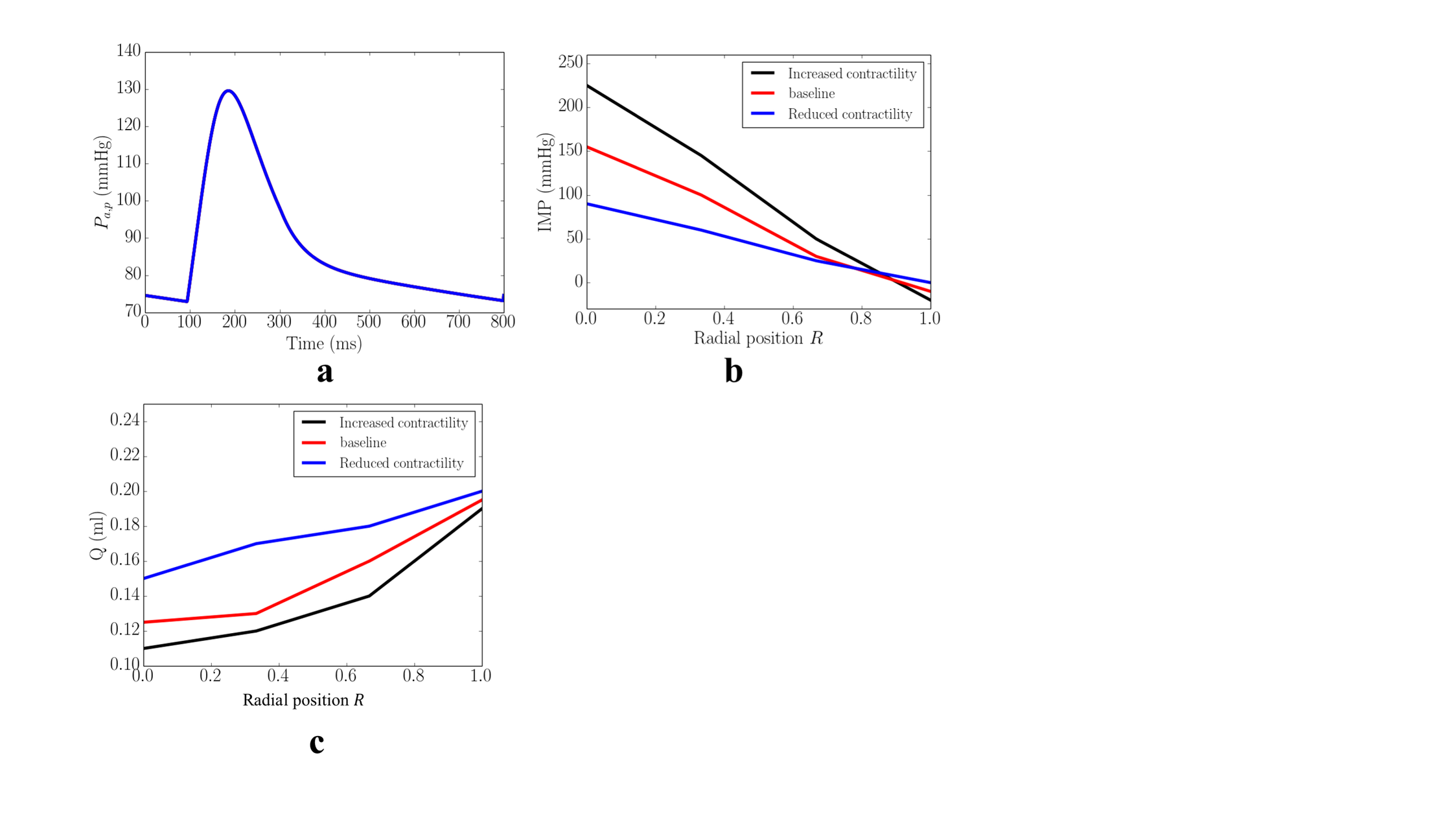
**

**Fig E1**

**
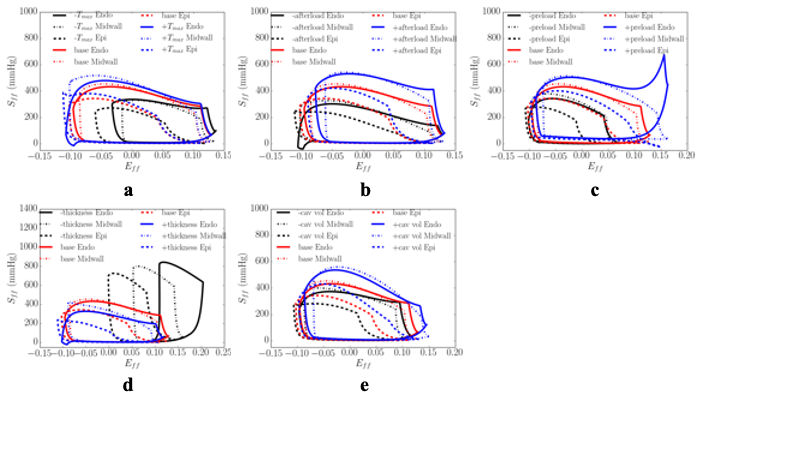
**

**Fig F1**

**
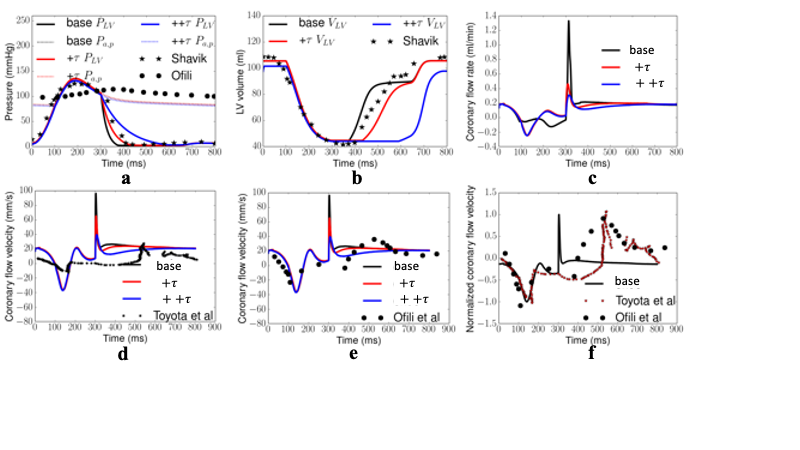
**

**
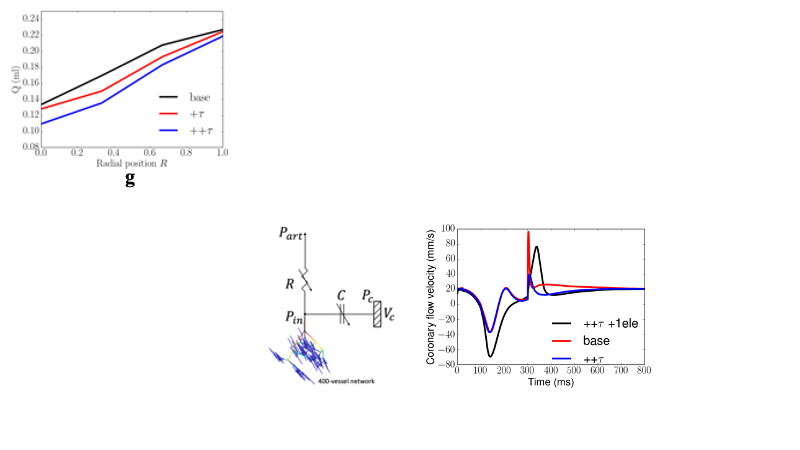
**

**Fig G1**

**
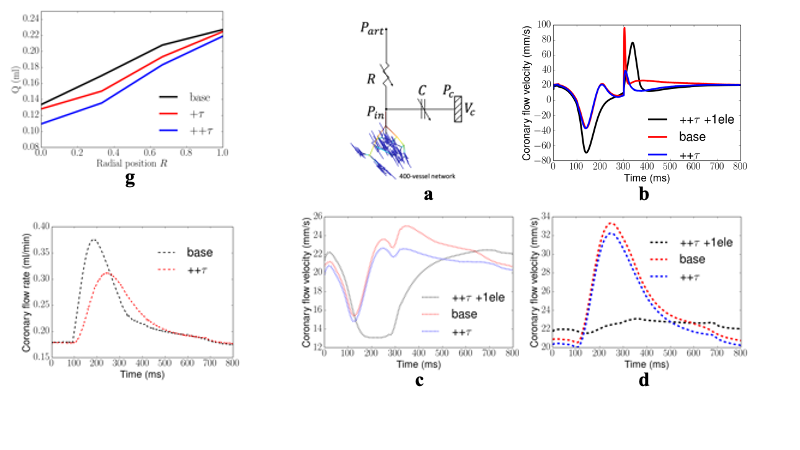
**

**Fig G2**

**
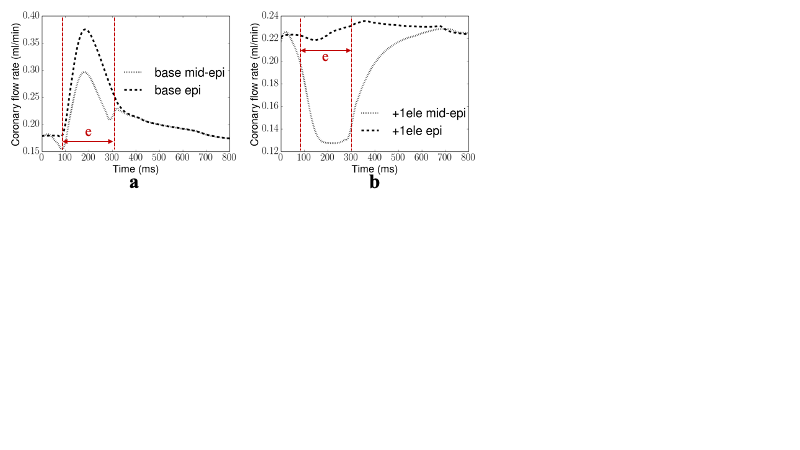
**
